# The Establishment and Application Studies on Precise Lysosome pH Indicator Based on Self-Decomposable Nanoparticles

**DOI:** 10.1186/s11671-020-03367-0

**Published:** 2020-07-08

**Authors:** Cui Pang, Chaojun Song, Yize Li, Qiaofeng Wang, Xiaosheng Zhu, Jianwei Wu, Yi Tian, Hao Fan, Jinwei Hu, Chen Li, Baolong Wang, Xiaoye Li, Wenchao Liu, Li Fan

**Affiliations:** 1 Department of Oncology, Xijing Hospital, Air Force Medical University, Xi’an, 710032 Shaanxi China; 2 School of Life Science, Northwestern Polytechnic University, 127th Youyi West Road, Xi’an, 710072 Shaanxi China; 3 Department of Pharmaceutical Analysis, School of Pharmacy, Air Force Medical University, Xi’an, 710032 Shaanxi China

**Keywords:** MB@SiO_2_, BPSi, Lysosome pH indicator, Autophagy

## Abstract

Acidic pH of lysosomes is closely related to autophagy; thus, well known of the precise lysosomes, pH changes will give more information on the autophagy process and status. So far, however, only pH changes in a relatively broad range could be indicated, the exact lysosomes pH detection has never arrived. In our study, we established an endo/lysosome pH indicator based on the self-decomposable SiO_2_ nanoparticle system with specific synthesis parameters. The central concentrated methylene blue (MB) in the central-hollow structural nanoparticles presented sensitive release as a function of pH values from pH 4.0–4.8, which is exactly the pH range of lysosomes. The linear correlation of the optical density (OD) values and the pH values has been built up, which has been used for the detection of lysosomes pH in 6 different cell lines. Moreover, by this system, we succeeded in precisely detecting the pH average changes of lysosomes before and after black mesoporous silicon (BPSi) NP endocytosis, clarifying the mechanism of the autophagy termination after BPSi endocytosis. So, the self-decomposable nanoparticle-based luminal pH indicator may provide a new methodology and strategy to know better of the lysosome pH, then indicate more details on the autophagy process or other important signaling about metabolisms.

## Introduction

Lysosomes serve as the final destination for macromolecules, where these macromolecules are degraded by hydrolytic enzymes activated by low pH [1]. The acidic pH of lysosomes maintained by the vacuolar-type H + −ATPase (v-ATPase) [2] that pumps protons from the cytoplasm into the lysosomal lumen was to keep the activity of ~ 60 types of hydrolytic enzymes [3]. Moreover, recent literature reports revealed that acidic pH of lysosomes is closely related to the autophagy [4], so that well known of the precise lysosome pH changes will give more information on the autophagy process and status. Based on our studies and literature review, amine-positive charged nanoparticle endocytosis will probably increase the pH change in endo/lysosomes, such as primary and secondary amine PEG-decorated nanoparticles or some hydrophilic decoration on the particle surface [5, 6].

The increase pH induced by the endocytosis of amine nanoparticles will dramatically increase in transcription factor EB (TFEB) nuclear localization [7], results in not only transcriptional upregulation of the pathway, but also causes lysosomal dysfunction, ultimately resulting in blockage of autophagic flux [7–9]. As TFEB regulates autophagy, and consequently, its overexpression leads to a significant increase in autophagosome production in cultured cells.

Thus, in order to predict the autophagy process and the details of autophagy, the lysosome precise pH and its change measurement are very crucial. Till now, from the endo/lysosome pH value indicating literature reviews [10] and the commercial products for detected the endo/lysosome pH values, only pH changes in a relatively broad range could be indicated, and the exact lysosome pH detection has never arrived. Thus, to know the insight details of autophagy, the establishment of a precise luminal pH change detection method is an important approach.

**Scheme 1:**
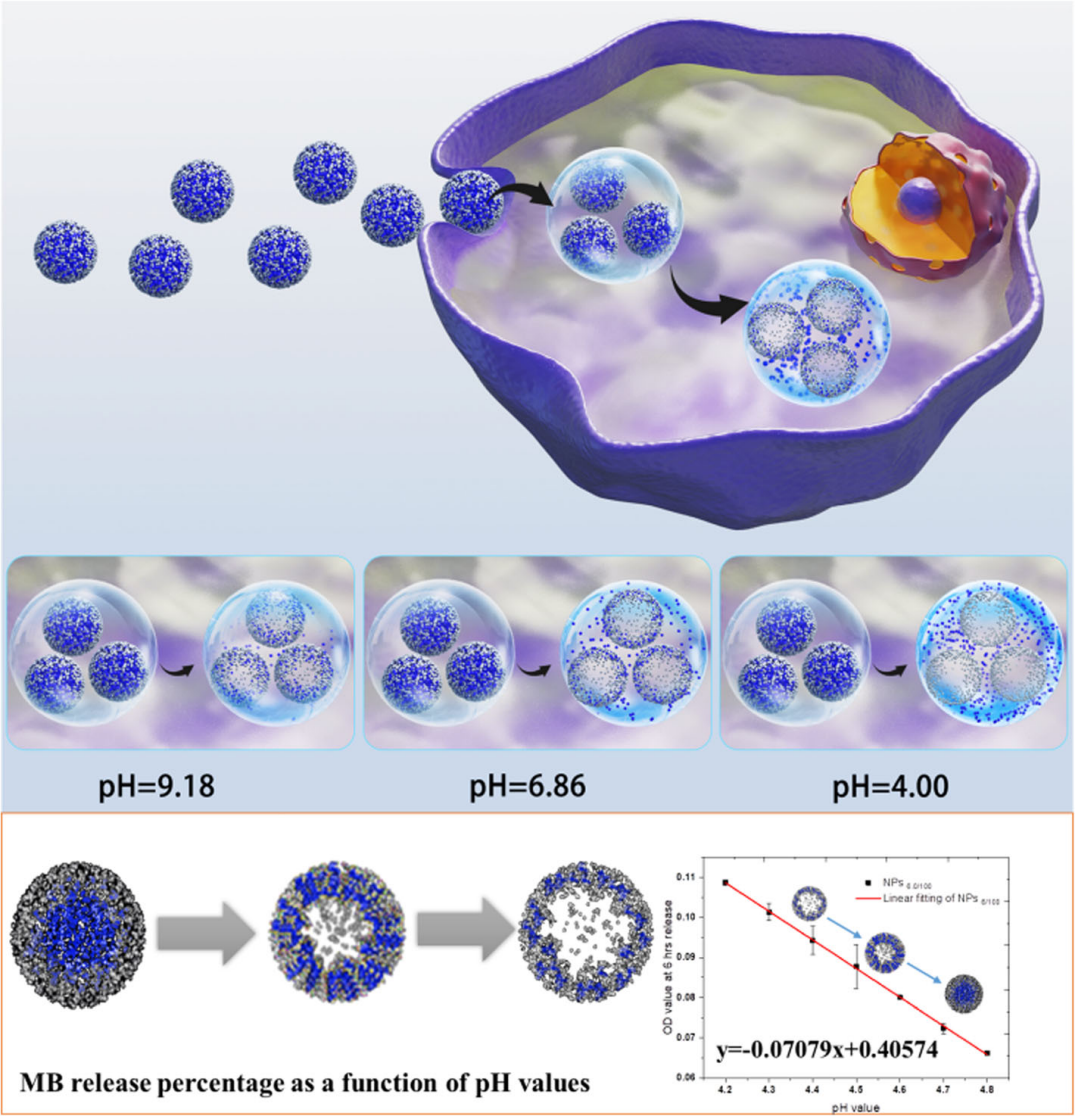
Schematic illustration of MB@SiO_2_ measurement of lysosome pH in living cells

Based on our previous experiences on self-decomposable SiO_2_ nanoparticles, in this study, we established a precise pH indicator which could realize the luminal pH changes detection. SiO_2_ nanoparticles have good advantages in tunable size and biocompatibility [11]. By setting specific synthesis parameters, the established self-decomposable SiO_2_ pH indicator could sensitively release the payload methylene blue (MB) in pH 4.0–4.8, which is exactly the pH range of lysosomes. Moreover, the MB release presented a linear correlation with the pH value changes (scheme 1). Then, we tested the feasibility of the pH indicator on cell levels by introducing 6 different cell lines, succeeded in determining the average pH changes of lysosomes before and after black mesoporous silicon (BPSi) NPs endocytosis, clarifying the mechanism of the autophagy termination after BPSi endocytosis. So, the self-decomposable nanoparticle-based luminal pH indicator may provide a new methodology and strategy to know better of the lysosome pH, then indicate more details on the autophagy process or other important signalings about metabolism.

## Materials and Methods

### Material Section

Sodium silicide (NaSi) and Si wafers (diameter 20 cm, p + (100), 0.01–0.02 Ω cm) were provided by SiGNa Chemistry Inc. and Ocmetic Inc., respectively. Ammonium bromide (NH_4_Br, 99%), sodium bromide (NaBr, 99%), toluene (anhydrous, 99.8%), hydrochloric acid (HCl, 37%), MB, and tetraethyl orthosilicate (TEOS) were bought from Sigma-Aldrich. 0.5 kDa methoxy-PEG-silane and 2 kDa methoxy-PEG-silane were bought from the Fluorochem Ltd. and Laysan Bio Inc. separately. RPMI 1640 medium was provided by the Life Technologies. Fetal bovine serum (FBS) was bought from TianHang biological technology. Sodium bicarbonate, streptomycin sulfate, penicillin G, HEPES, Lysozyme Solution, CellLight Early-endosomes-GFP, LysoTracker™ Red DND-99, Pierce® BCA Protein Assay Kit, enhanced chemiluminescence, pHrodo™ Red Transferrin conjugate, Live Cell Imaging Solution, and Trizol reagent were bought from Thermo Fisher Scientific. Ethanol and ammonia–water were provided by the Sinopharm. BioRT Master HiSensi cDNA First-Strand Synthesis kit was bought from the Hangzhou Bioer Technology Co., Ltd. RIPA lysate was bought from the Heart Biological Technology Co., Ltd. P62, TFEB, and β-actin antibody were bought from the Proteintech Group, Inc. LC 3B antibody was provided by the Abcam. 2-(4-Pyridyl)-5-((4-(2-dimethylaminoethy-laminocarbamoyl) methoxy) phenyl) oxazole (PDMPO) was provided by the Yeasen Biotech Co., Ltd.

### Aim, Design, and Setting of the Study

The aim of this study is to (1) explore the influence of BPSi nanoparticles on autophagy in HepG2 cells, (2) find out the underneath mechanism of lysosome pH value changes affecting autophagy, (3) establish a precise lysosome pH indicator which could measure the pH of lysosomes precisely, and finally, (4) indicate the influence of pH fluctuation on autophagy. In order to realize the study purpose above, we employed the transcriptome sequencing experiment to explore the transcriptome gene changes in HepG2 cells after feeding BPSi nanoparticles and verified it by RT-qPCR and Western experiments. Fluorescent dyes such as PDMPO were used to measure the change of lysosomal pH in HepG2 cells after feeding BPSi. To measure the pH of lysosomes precisely, we developed MB@SiO_2_ nanoparticles with 10 parameters and tested the characterizations of these 10 kinds of nanoparticles through experiments such as DLS and HR-TEM. The MB loading efficiency and release kinetics study of 10 series self-decomposable nanoparticle systems were tested in different pH solutions and HepG2 cells. To verify the nanoparticle intracellular location after entering the cell, we did cell TEM experiment and live-cell confocal microscopy. Finally, we measured the lysosomal pH changes in 6 kinds of cells after feeding BPSi to verify the universality of MB@SiO_2_ nanoparticles to measure the lysosomal pH changes.

### BPSi Nanoparticle Synthesis

The BPSi nanoparticles were prepared by our previous method [12] and supplied by our collaborator (Wujun Xu, Department of Applied Physics, University of Eastern Finland). The preparation of BPSi, NaSi, ammonium salt, and NaBr (NaSi: NH_4_Br: NaBr of 1:4:4, w/w/w) was ground in a glove box with an Ar atmosphere. They were allowed to react in a tube oven under an N_2_ atmosphere at 240 °C for 5 h (Eq. 1). After being cooled down to ambient temperature, the obtained microparticles were purified by rinsing with 0.5 M HCl and 1.0 M HF solutions separately. The microparticles were ball-milled in ethanol at 1000 rpm for 15 min, and the BPSi nanoparticles with the desired diameter were collected via adjusting the centrifugation speed.

1 $$ \mathrm{NaSi}+{\mathrm{NH}}_4\mathrm{Br}\to \mathrm{NaBr}+{\mathrm{NH}}_3+\mathrm{Si}/\mathrm{H}+{\mathrm{H}}_2 $$

Through the Dynamic Light Scattering experiment, the diameter distribution and surface charge of the nanoparticle were studied. All of the NPs were dispersed in the medium after sterilization by slight ultrasonication (5 s to make them evenly dispersed in the solution, Ultrasonic cleaner SB-5200DT, Ningbo Scientz Biotechnology Co., Ltd.) right before their introduction to the cells.

### 10 Series Self-Decomposable Nanoparticle System Establishment

The 10 series self-decomposable nanoparticles were synthesized by the methodologies we reported before [13–16], with modified parameters. In a typical procedure, a certain amount of MB was firstly added to a mixture of ethanol (75 mL) with ammonia–water solution (25%, 3.4 mL), after that certain amount of TEOS was added. The series self-decomposable MB@SiO_2_ NPs were obtained after stirring for 24 h and washed 3 times before their being dried. The MB and TEOS amounts added in the protocols were as described in Table 1. The meaning of 1.0/100 in the NPs_1.0/100_ represented the inventory of MB and TEOS when we synthesized the nanoparticles, with 1.0 mg of MB and 100 μL of TEOS. And the meanings of 1.5/100 in the NPs_1.5/100_ and others are consistent with that of 1.0/100.

**Table 1:** MB and TEOS amounts added in the reaction solution

Groups	MB amount	TEOS amount (μL)
NPs_1.0/100_	1.0	100
NPs_1.5/100_	1.5	100
NPs_3.0/100_	3.0	100
NPs_4.0/100_	4.0	100
NPs_6.0/100_	6.0	100
NPs_1.5/80_	1.5	80
NPs_2.0/80_	2.0	80
NPs_2.5/80_	2.5	80
NPs_5.0/80_	5.0	80
NPs_7.5/80_	7.5	80

### Cell Culture

In order to test the efficiency and universality of the pH indicator based on self-decomposable nanoparticles, we tried to test it on specific tumor-derived cancer cell lines. Thus, we selected liver cancer, lung cancer, colon cancer, and melanocytoma cell lines as the research objects. The cell lines of human colon cancer cells HCT116, HCT8, and HCT15; human liver cancer cells HepG-2; human lung cancer cells A549; and mouse melanoma cells B16 were maintained in RPMI 1640 medium (Life Technologies) supplemented with 10% heat-inactivated FBS, 2.0 g/L sodium bicarbonate, 0.1 g/L streptomycin sulfate, 0.06 g/L penicillin G, and 5.958 g/L HEPES. The cells were maintained in a standard, cell culture incubator at 37 °C in a humidified atmosphere with 5% CO_2_.

### Characterizations of 10 Series Self-Decomposable Nanoparticle Systems

The morphology of all series nanoparticles was characterized by HR-TEM with STEM mode, and Si mapping was studied by EDS element mapping. Nanoparticle size distribution analysis was performed by ImageJ software by calculating the nanoparticle diameters in randomly selected STEM images. The zeta potential of the nanoparticles and the polydispersity index (PDI) has been measured by the dynamic light scattering (DLS) study, in series buffers with specific pH values. Data was analyzed by SPSS15.0 and the statistical results were presented as mean ± S.D.

### The MB Loading Efficiency and Release Kinetics Study of 10 Series Self-Decomposable Nanoparticle Systems

In order to study the MB loading efficiency and release kinetics, the standard curve of MB in series concentrations was first established. The absorption of MB was carried out by UV–Vis spectrum with the absorbance at 660 nm, which is the *λ*max of the monomer MB. The MB loading efficiency was calculated by the equation below, MB loading efficiency (%) = the amount of encapsulated MB/(total amount of MB input).

The MB release from 10 series nanoparticles was studied in pure water and pH buffers with different pH values (pH 4.0, pH 6.86, and pH 9.18) and Lysozyme Solution (Thermo Scientific™ ^#^90082). Moreover, the MB release kinetics after a specific duration in different pH buffers were also investigated. The OD values at 660 nm and the MB release percentages as a function of time were then studied.

In more details, the MB release studies were performed by the protocols below; dissolved 10 series nanoparticles in 15 mL standard buffer of pH 4.0, 6.86, and 9.18 with lysosome solution, respectively; and performed the MB release in a Hula mixer at 37 °C. During the following 15 days, 1 mL of each sample was collected, then centrifuged at 12000 rpm for 10 min. The supernatant and the precipitate were measured for their absorption spectra at 200–800 nm.

Moreover, the MB release in precise pH buffers with lysozyme solution in the pH range from 4.1 to 5.5 was also investigated with the same protocols above. Specific time durations (6 h, 12 h, and 24 h) were placed as observation time points. The absorptions at 660 nm were recorded in each sample. The linear relationship of absorption of each pH solution and the sum of the squares of the residuals were counted at each time point, respectively.

To detect the MB release profiles in cells, HepG-2 cells were cultured in a 75 cm^2^ culture flask and fed with NPs (300 μg/mL) when the cells were proliferated to 70% of the culture flask. Every 30 min later, the cells were collected. The cells were repeatedly frozen and thawed to release the MB in the cells completely. The cell lysates were centrifuged at 12000 r/min for 10 min. The supernatant was obtained and measured its absorbance at 660 nm to calculate the total amount of released MB. In this study, HepG2 cells were chosen as the research objects, due to their quick cell proliferation which could minimize the variance among all 10 tested groups.

### Cellular Colocalization of the 10 Series Nanoparticles and the Release Performance in 6 Different Cell Lines

Cell TEM was employed to study the colocalization of the nanoparticle in the endo/lysosomes following standard cell TEM protocols. Cells were seeded in the intensity of 1 × 10^6^ cells/flask and incubated for 24 h, allowing the cell attachment. Ten series of nanoparticles in medium with the same concentration (100 μg/mL) were incubated with the cells for another 12 and 24 h, respectively. Cells were then washed by PBS for 3 times to remove the excess nanoparticles, then fixed in 2.5% glutaraldehyde solution for longer than 1 day. Fixed cells were then washed and stained by osmium tetroxide, 1% in deionized water for 1 h, followed by washing with PBS for 3 times and DI water for 2 times. Classic cell TEM protocol [17, 18] was carried out next, and sections with a thickness of 90 nm were collected for TEM observation. MB release as a function of pH values was studied in 6 cell lines with both NPs_6/100_ and NPs_7.5/80_. Also, the OD values from the release of the MB and the MB release percentages were recorded for data analysis.

### Investigation of Intracellular Uptake of MB@SiO_2_ Nanoparticles

Live-cell confocal microscopy was used to assess the cellular uptake and intracellular fate of the MB@SiO_2_ nanoparticles. HepG-2 cell early endosomes were stained (CellLight Early-endosomes-GFP, BacMam 2.0 ThermoFisher Scientific C10586, with excitation/emission ~ 488/510 nm) for 16 h. And then cells were incubated with NPs_6/100_ (MB excitation/emission: 640/650–700 nm) at the nanoparticle concentration of 100 μg/mL at specific time intervals (2 h, 2.5 h, 3 h, 5 h, and 6 h). Before images were taken, lysotracker was stained with the LysoTracker™ Red DND-99 (Thermo Fisher Scientific L7528, excitation/emission: 577/590 nm) for 40 min. After that, remove the stain solution and wash the cells 2–3 times in PBS. Images were taken using a Nikon A1R Confocal Microscope.

### Transcriptome Sequencing to Evaluate the Gene Expression Change after BPSi Feeding

Total RNA extraction of the control group and BPSi-treated group was performed using the Trizol reagent following the standard operating procedures. The quality of the initial total RNA sample for the sequencing experiment was detected using a NanoDrop ND-2000 spectrophotometer. Total RNA that passed the quality control was used in subsequent sequencing experiments. A comparison of the gene expression was performed by next-generation sequencing. All sequencing programs were performed by BGI-Shenzhen Corporation (Shenzhen, China) using the BGISEQ-500 platform. Raw data obtained by sequencing are performed quality control to determine whether the sequencing data is suitable for subsequent analysis. If passed, perform quantitative analysis of genes based on gene expression levels and perform significant enrichment analysis of gene ontology (GO) functions on the differentially expressed genes between the selected samples.

### Reverse Transcription Quantitative Polymerase Chain Reaction (RT-qPCR) Assay to Confirm the Activation of TFEB-CLEAR Gene Network

Total RNA was extracted from the cultured HepG-2 cells of the control and BPSi-treated groups by using the Trizol reagent and reverse-transcribed to cDNA by using the BioRT Master HiSensi cDNA First-Strand Synthesis Kit (Hangzhou Bioer Technology Co., Ltd.) with random primers. cDNA was used to amplify TFEB-CLEAR gene network by quantitative PCR with the Applied Biosystems™ 7500 real-time PCR system (Applied Biosystems, Life Technologies, Carlsbad, CA) with actin as a reference control. Primers used for quantitative RT-PCR were listed in Table S4.

### Western Blot Assay to Confirm that Autophagy Is Activated after BPSi Feeding

Cellular proteins of the control group and different concentration BPSi-treated groups were extracted by RIPA lysate (Heart Biological Technology Co., Ltd.). The protease inhibitor was added to the RIPA lysate and pre-cooled on ice. Washed the cells 3 times by pre-cooled PBS. Dumped the liquid completely and placed the dish in ice for 2 min. Four hundred microliters of RIPA lysate was added to the surface of the whole dish, pipetted several times with a pipette, and incubated on ice for 30 min, during which the dish was shaken several times to completely lyse the cells. The lysed cell fluid was transferred to 1 ml Eppendorf tubes and centrifuged at 13,000 rpm for 10 min, 4 °C. The obtained supernatant was boiled in water for 10 min and placed in – 20 °C for later use. Protein concentration was quantified using the Pierce® BCA Protein Assay Kit (Thermo scientific).

Cell extracts containing 25 μg total protein were directly subjected to SDS-PAGE and transferred. The membranes were blocked with 5% skimmed milk and probed with primary antibodies that recognize P62 (Proteintech ^#^18420–1-AP), TFEB (Proteintech # 13372–1-AP), LC 3B (Abcam ^#^ ab192890), and β-actin (Proteintech ^#^20536–1-AP). Secondary antibodies were chosen according to the species of origin of the primary antibodies and detected by enhanced chemiluminescence (Pierce) or by using the Bio-Rad ChemiDoc XRS + Gel Imaging System (Bio-Rad, USA). The normalized band intensity of P62, TFEB, and LC 3B relative to β-actin was quantified by densitometry using ImageJ software in the BPSi groups, and the data are the mean ± S.D. from three independent experiments.

### Measuring Cell pH by PDMPO and pHrodo™ Red Transferrin Conjugate by Confocal Microscopy

#### PDMPO Study

1 × 10^5^ HepG-2 cells were cultured on sterile confocal plates overnight and the BPSi nanoparticles were fed with a concentration of 100 μg/mL. The next day, before immunofluorescence staining, slides were washed three times with 0.01 M phosphate-buffered saline (PBS), pH 7.4, then added 1 μM PDMPO dye (Ex/Em = 329/440). After washing with PBS for three times, cells were incubated with fresh RPMI-1640 culture medium and observed under a fluorescence microscope (Nikon A1R, Japan) with a CCD camera and take pictures within 5 min, and the ratio of blue and green fluorescence intensity in the lysosomes was then calculated according to the procedure of Chen et al. [19].

#### pHrodo™ Red Transferrin Conjugate Study

HepG-2 cells were plated in confocal plates in the same way for cell attachment for 24 h, then kept plates on ice for 10 min. Washed cells with cold Live Cell Imaging Solution containing 20 mM glucose and 1% BSA. Added pHrodo™ Red Transferrin conjugate (Ex/Em = 560/585 nm) at 25 μg/mL in Live Cell Imaging Solution and incubate at 37 °C for 20 min, then washed cells in Live Cell Imaging Solution. The observation was also carried out by confocal microscopy. The quantitative analysis of the intensity of the microscopy images was performed by ImageJ software.

#### Detection of Cell Lysosomal pH

The cells A549, HepG-2, HCT8, HCT15, HCT116, and B16 were cultured in a 75 cm^2^ culture flask and fed with NPs when the cells were proliferated to 70% of the culture flask, and 6 h later, the cells were collected. The cells were repeatedly frozen and thawed to release the MB in the cells completely. The cell lysates were centrifuged at 12000 r/min for 10 min. The supernatant was obtained and measured its absorbance at 660 nm to calculate the total amount of released MB. The absorbance of the NPs_6.0/100_ at a standard pH was compared to obtain the pH value of each cell.

### Statistical Analysis

Statistical analysis was done with the SPSS15.0 software by using a two-way analysis of variance (ANOVA) for independent groups and using Tukey HSD method for multiple comparisons test. Statistical significance was based on a value of *P* < 0.05.

## Results and Discussion

**Fig. 1:**
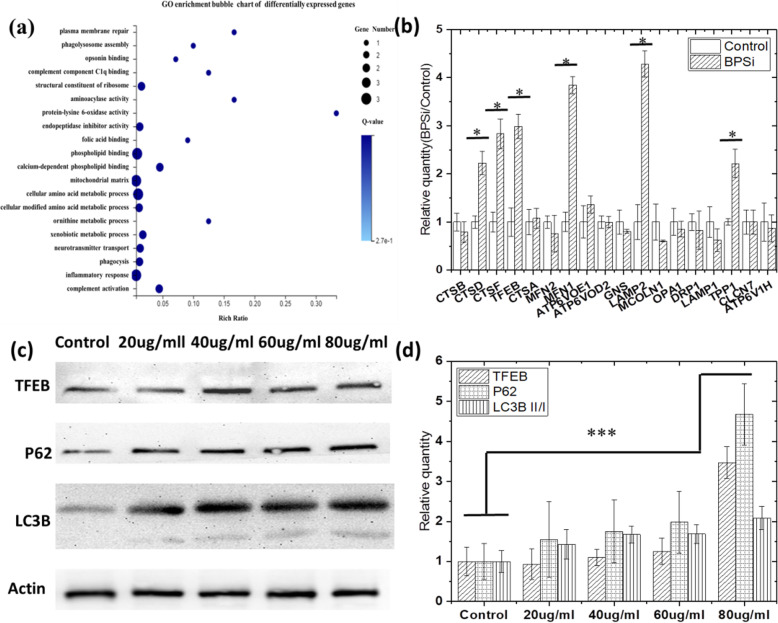
Autophagy is activated in HepG-2 cells after feeding BPSi. **a** GO enrichment bubble map of the differentially expressed genes found by transcriptome sequencing. **b** Verification of gene changes in the TFEB-CLEAR gene network after BPSi treated by RT-qPCT experiments. **c** P62, TFEB, LC3B II/I protein expression after BPSi-treated. **d** Normalized band gray intensity of P62, TFEB, LC3B II/I protein in BPSi-treated groups according to the control group. Data was presented as mean ± S.D.

We firstly detected differential gene expression when cells fed with the dual PEG functional black porous silica nanoparticles (BPSi NPs) (offered by our collaborating lab [12]). The change of the zeta potential from − 18.5 to + 2.8 mV also indicated that the surface dual-PEGylation was successful (Fig. S1a). The mean diameter of BPSi nanoparticles was 156 nm (Fig. S1b). Based on the cluster heat map of differential gene expression (Fig. S2), we selected more than 2-fold differential gene expressions for further investigation. Go and KEGG was introduced to analyze the differential genes. From the Go enrichment bubble maps (Fig. 1a), the metabolic and lysosome-associated genes including phagolysosome assembly, phagocytosis, and xenobiotic metabolic process were selected for further analysis. Notably, TFEB-CLEAR [17]-associated gene expressions were significantly increased. The RT-PCR results (Fig. 1b) also verified the gene sequencing results, the genes on TFEB-the coordinated lysosomal expression, and regulation (CLEAR) pathway significantly increased, such as CTSD, CTSF, TFEB, MFN1, LAMP2, and TPP1. These genes have been marked on the lysosome pathway, as shown in Fig. S3. Their expression is higher than that of the control group and has statistical significance (*P*_BPSi VS Control_ < 0.05). And TFEB positively regulates the expression of lysosomal genes, controls the lysosome population, and promotes cellular degradation of lysosomal substrates.

Additionally, TFEB regulates autophagy, and its overexpression leads to a significant increase in autophagosome production in cultured cells due to the fact that the major function of TFEB gene is to induce the bio-synthesis of lysosome and promote the occurrence of autophagy [20]. Western blot analysis was also employed to testify whether the autophagy happened when cells fed with BPSi NPs. The purpose of the Western test was to further confirm that the expression of TFEB was increased and the occurrence of cell autophagy after BPSi feeding. The LC3B and P62 proteins are both autophagy markers. When autophagy occurs, the expression of microtubule-associated protein 1A/1B-light chain 3B (LC3B) II/I increases. p62 is a receptor for vesicles that will be degraded by autophagy and is also a receptor for ubiquitinated protein aggregates to be cleared, and its expression decreases when autophagy occurs. So we measure the expression of these proteins in the Western experiment.

From the Western blot results shown in Fig. 1c, d, TFEB (*P*_80 μg/ml VS control_ = 0.000008), LC3B II/I (*P*_80 μg/ml VS control_ = 0.000297), and p62 (*P*_80 μg/ml VS control_ = 0.000016) proteins all significantly upregulated. As the upregulation of TFEB and LC3B II/I proteins indicating the activation of autophagy [18], we suspected that the BPSi endocytosis promotes the occurrence of autophagy. However, the p62 protein is supposed to be downregulated during the autophagy process, due to the carrier protein nature which brought the endosomes to lysosomes and finally degraded. In our study, the significant upregulation of p62 indicated the termination of degradation during the endo-lysosome fusion process [21], which probably caused by pH increasing in endo-lysosome vesicles. Thus, the BPSi endocytosis may firstly induce the occurrence of autophagy, then inhibited the autophagy process by increasing the endo/lysosome pH values, owing to its amide alkalinity.

In order to testify the pH increase characteristics in endo/lysosomes by BPSi endocytosis, two commercial pH fluorescent probes were employed in our study, pHrodo™ Red Transferrin Conjugate (Thermo Fisher ^#^P35376), and RatioWorks™ PDMPO.

pHrodo™ Red as a commercial intracellular pH indicator usually presents weakly fluorescent at neutral pH but increasing fluorescent as the pH drops. It was supposed to quantify cellular cytosolic pH in the range of 9–4 with a pKa of ~ 6.5 with excitation/emission of 560/585 nm. We could obtain a qualitative analysis conclusion from 6 cell line determinations that the endocytosis of BPSi NPs has the capability of increasing the pH values in endo/lysosomes, due to the weakening red fluorescent signals (Fig. S4 and S5). However, after repeating the experiments several times following the product operation protocols, we hardly quantitatively analyzed the exact pH value that decreased among different cell lines before or after feeding with BPSi NPs due to having no correlation between intensity and the pH values established.

PDMPO was then employed as a better solution for indicating the pH value changes after BPSi endocytosis, which introduce ratio imaging technics in pH quantitative measurement. PDMPO [2-(4-pyridyl)-5-((4-(2-dimethylaminoethy-laminocarbamoyl) methoxy) phenyl) oxazole] is characterized as acidotropic dual-excitation and dual-emission pH probe. It emits intense green fluorescence at lower pH and gives intense blue fluorescence at higher pH. This unique pH-dependent fluorescence makes PDMPO an ideal pH probe for acidic organelles with pKa = 4.47. PDMPO selectively labels acidic organelles (such as lysosomes) of live cells and the two distinct emission peaks can be used to monitor the pH fluctuations of live cells in ratio measurements. However, we still failed in measuring the pH values in 6 cell lines before and after BPSi feeding. As the results shown in Fig. S6, no significant differences were observed in all 6 cell lines before and after BPSi feeding. Though a correlation has been established between the Blue/Green Ratio and the pH values (Fig. S7), nonlinear correlation from pH 4–5 makes the PDMPO method failed in quantitative analysis of endo/lysosomes before and after BPSi feedings.

From the data of two commercial pH indicators above, we firstly demonstrated our suspect that PEG-decorated nanoparticles with amide on the PEG chain could make the endo/lysosome pH increase due to the alkaline nature of amide. However, without the quantitative analysis of precise pH changes (0.1 pH range), we still cannot establish the correlations between the autophagy status and the endo/lysosome pH values, thus failed in autophagy prediction.

Based on our previous study on self-decomposable nanoparticles [13, 14] [15, 22], we kept the same concentration of ammonium hydroxide in 75% ethanol, but adjusted the MB and TEOS concentrations. Two series of TEOS amount have been set as 100 μL and 80 μL, in order to obtain different shell thickness and pore size. Ten series of MB amount have been set to obtain different sizes of the center-hollow structure and MB loading efficacies.

The MB and TEOS amounts added in the protocols were as described in Table 1 below.

**Fig. 2:**
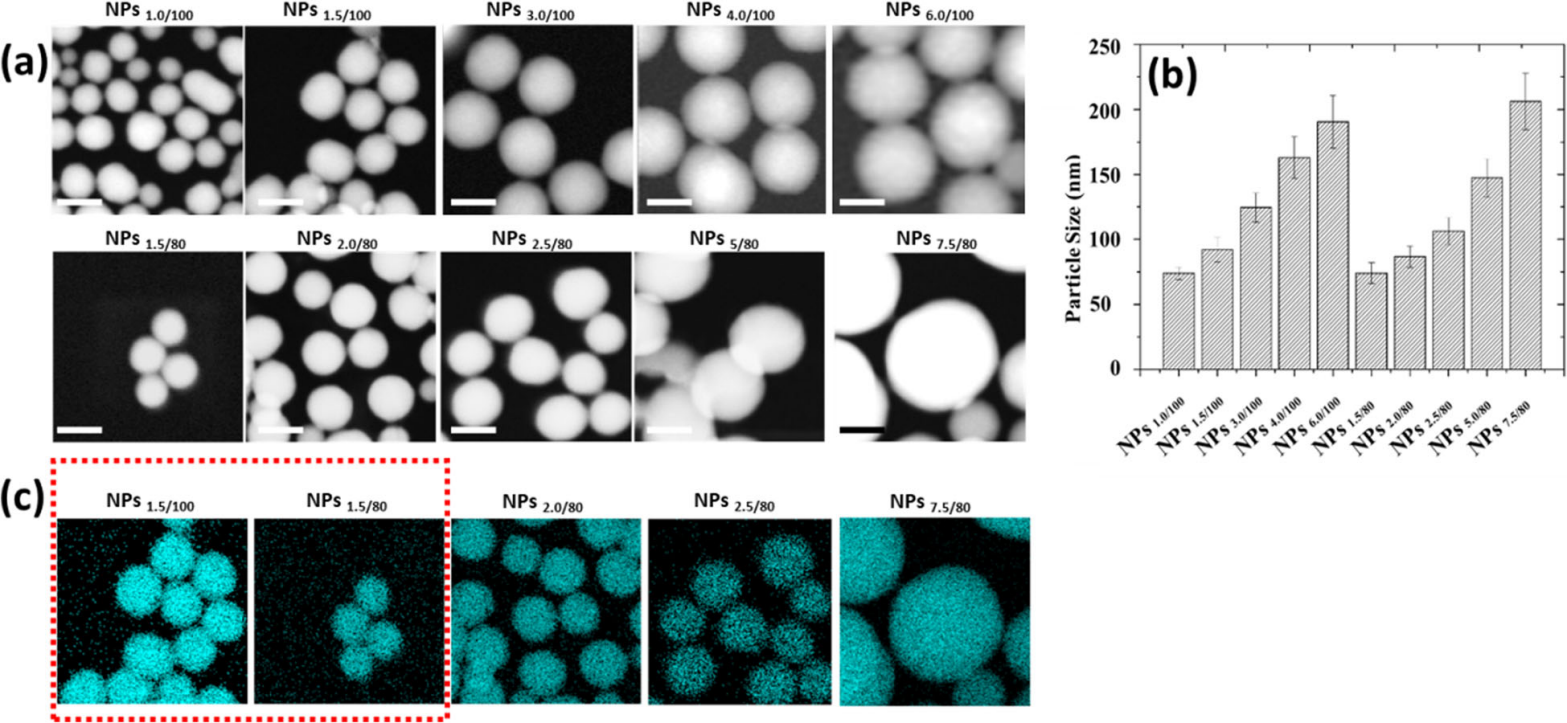
Morphology characterization of 10 different self-decomposable nanoparticles with specific MB or TEOS amount. **a** STEM figures. **b** Nanoparticle size distribution analysis. **c** Si mapping of 10 self-decomposable nanoparticles. Scale bars in all figures are 100 nm. The size distribution analysis was performed by randomly chosen 100 nanoparticles from STEM figures and measured by ImageJ software. Data was presented as mean ± S.D.

As shown in Fig. 2a, b, the nanoparticle size increased with the increase of the MB amount, in both TEOS concentrations (100 μL and 80 μL). At the same MB concentration, the particle size increased with the increase of TEOS amount. Moreover, with the increase of the TEOS amount, the shell thickness grew up, which has been proved by the element mapping (shown as Fig. 2c). The polydispersity index (PDI) and surface charge of the nanoparticles are shown in Fig. S8 and Table S1. The morphology studies predicted that with the increase of MB amount, the loading efficiency will grow up, leading to the faster release profile, while with the increase of TEOS amount, the release will slow down. And we need to find out the appropriate MB and TEOS concentration, with which we could obtain the optimized nanoparticle systems, that we may be able to make the MB release profile linear correlated with the pH changes.

The MB loading efficiency was determined by UV-Vis spectrum. The standard curve (Fig. S9) of MB was firstly drawn using series concentrations of MB solution (from 6.25 to 46.88 μg/mL), with the equation as *y* = 67.63*x* + 0.10919, *R*^2^ = 0.9987. As calculated with the equation above, we obtain MB loading efficiency of 10 self-decomposable nanoparticles with specific parameters, detailed data shown in Fig. S10.

Before the study of the MB release profiles in different pH solutions, the release profiles in pure water have been studied. As shown in Fig. S11 and Fig. S12, all the nanoparticles with TEOS amount of 80 μL presented increased MB release along with the duration increase, which was reflected by the UV–Vis absorption. Moreover, with the MB encapsulated amount increase, the growth trend of MB release becomes more significant. Also, the release velocity grows faster. However, as the TEOS amount increase to 100 μL, the particle surface became more densed and the release becomes slower when the MB amount below 3.0 mg; almost no increase trend could be observed in the MB release in water during 14 days of release. As long as the MB amount increases to above 4.0 mg, an obvious increase trend of MB release could be observed. One thing to be noticed is that the nanoparticle parameter of both NPs_7.5/80_ and NPs_6.0/100_ presented solid growth as the time prolongs, almost showed a linear increase trend during the first 7 days, and then reached the platform.

Then, we focused on the MB release behavior in different pH buffers to figure out whether self-decomposable nanoparticles with specific parameters could have the linear pH-dependent MB release.

Firstly, we carried out the MB release experiments at pH 4.0 buffer solution. From Fig. S13, we could easily reach the conclusion that with the same TEOS amount of 100 μL, the MB release velocity presented a similar trend was observed in the 5 nanoparticle systems of TEOS at 80 μL (Fig. S14), the center positive correlation with the MB encapsulated amount.

**Fig. 3:**
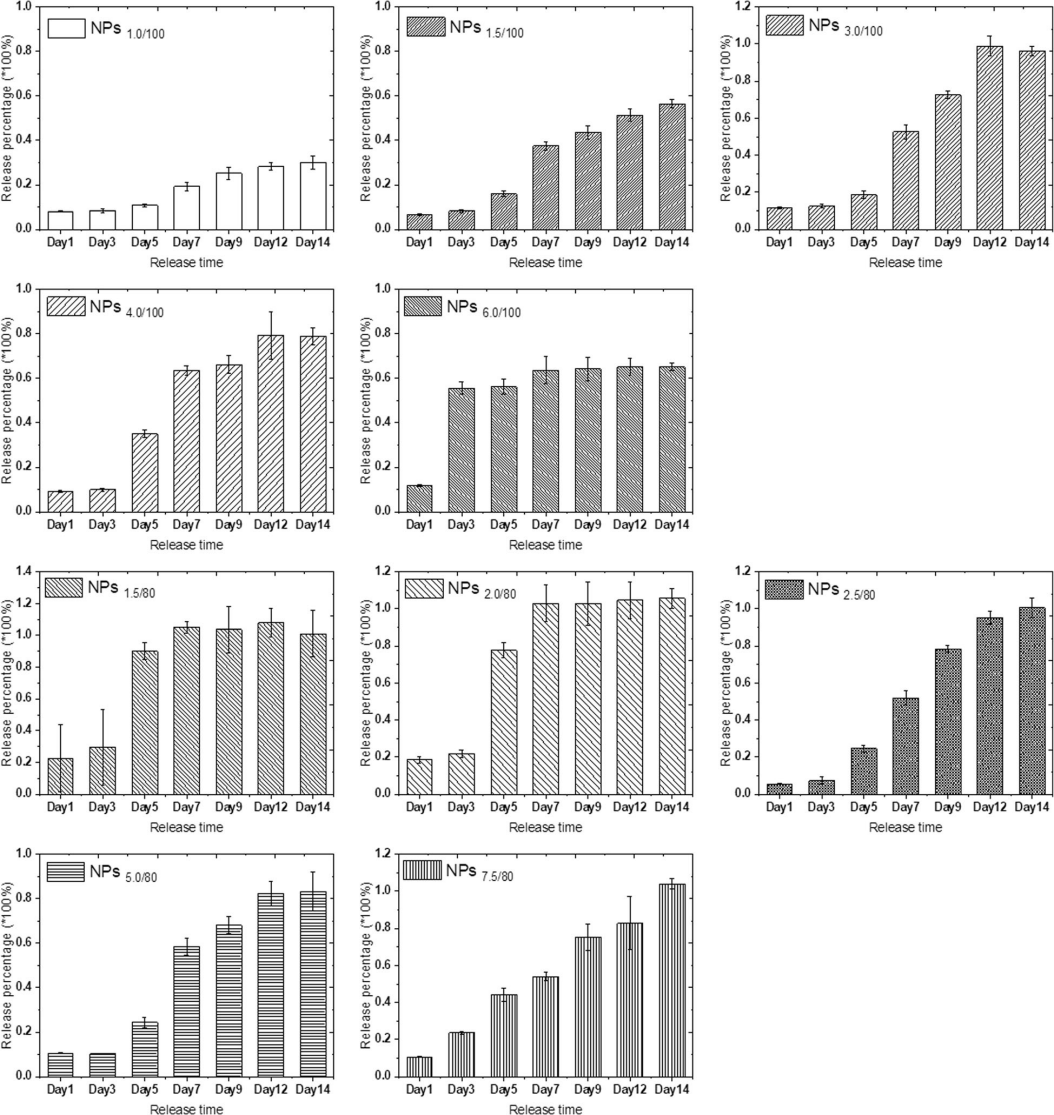
MB release percentage of 10 series self-decomposable nanoparticles after a specific duration in pH 4.0 buffer. All experiments were triple repeated, and the data were shown as mean ± S.D.

Concentrated MB diffuses into the surrounding solution via diffusion due to concentration difference. The bigger concentration gradient makes the faster MB release. Compared with the MB release in pure water, we found that the acidic environment speeded up the release of MB (Fig. S13 and S14 compared with Fig. S11 and S12), indicating that the MB release is not only driven by diffusion; however, in acidic solutions, electrostatic repulsion is also an important driven force due to the positive charge nature of MB. We then calculated the release percentage of each nanoparticle parameter according to the MB loading efficiency, MB standard curve, and the dilution ratio at measurements. The release percentage reflected the release speed of MB in pH 4.0 acidic solution, and the results (Fig. 3) showed that only the release percentage of NPs_7.5/80_ presented linear release in pH 4.0 solution. Other nanoparticle systems with specific MB and TEOS parameters showed similar release trends, and the release percentage did not have a linear growth. One exception is NPs_6/100_, and the MB release reached the platform in only 72 h; thus, it was hard to tell whether the MB release could grow linear before that duration at this stage.

Meanwhile, we tested the MB release profiles in near-neutral and alkali buffers (pH 6.86 and pH 9.18). The results in both Fig. S15, S16, Fig. S17, and S18 demonstrated that the MB release slowed down with the solution pH increase to 6.86; moreover, with the central MB concentration increased, the MB release percentage decreased. At pH 9.18, all nanoparticles with 10 specific parameters presented a very slow MB release (Fig. S19 and S20); no matter in UV–Vis absorption or the release percentage, the trend was similar with the one in pH 6.86 buffer, but with even lower release percentage. So, it was clear that the self-decomposable nanoparticles only presented MB release linear growth in acidic solutions. We thought back to the endo/lysosomes pH, from 4 to 5, which is exactly the pH range of MB linear growth as a function of time in a specific MB/TEOS parameter. Thus, we get more confident that the self-decomposable nanoparticle system may be an accurate measuring tool for quantitative determining the endo/lysosome average pH, then provide evidence on the exact pH value of autophagy status.

The precondition of using the specific self-decomposable nanoparticles as an endo/lysosome pH indicator is that the nanoparticles stay stable in the endo/lysosome during the whole measurement process. Secondly, the MB release in endo/lysosome should occur smoothly when the measurement carried out.

**Fig. 4:**
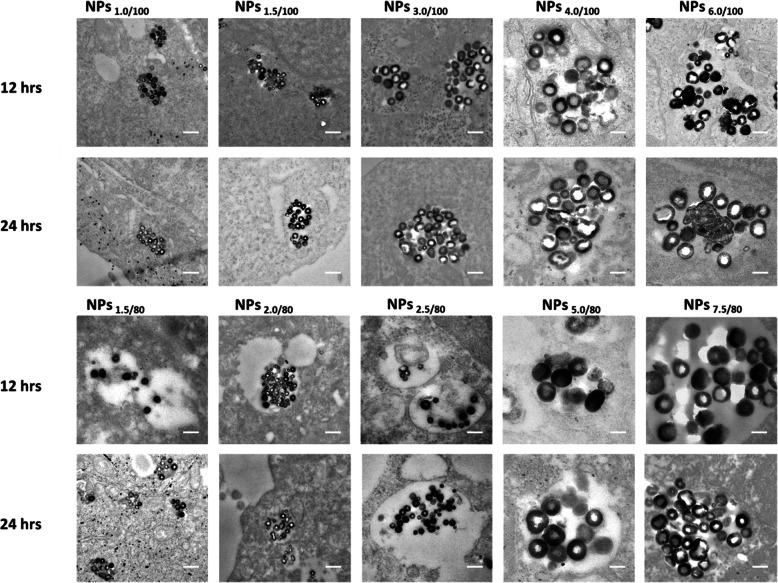
The colocalization of the nanoparticle in the endo/lysosomes by cell TEM study after 12 and 24 h incubation with the 10 series nanoparticles. The scale bar is 200 nm in the TEM images

The colocalization of the nanoparticle in the endo/lysosomes by cell TEM study and the MB release in 6 different cell lines were studied. From the cell TEM results, all of the 10 series nanoparticles stayed in the endo/lysosomes without escaping, after 24-h incubation with the HepG-2 cells (Fig. 4). Since the diameter of HepG2 cells used in Fig. 4 is about 10–20 μm and the diameter of MB@SiO_2_ nanoparticles is between 75 and 200 nm, it will be very difficult to clarify the nanoparticle morphologies using the images with low magnification (as shown in Fig. S21). We also investigated the intracellular location of the nanoparticles in the other 5 cell lines, and 4 nanoparticles were randomly selected to demonstrate the nanoparticles were trapped in the endo/lysosomes (Fig. S22). The nanoparticles with all parameters showed a central hollow structure in all other 5 cell lines after 24-h incubation, indicating the MB release. Moreover, under more precise observation, we noticed the MB release may be different due to different hollow sizes, pointing to the fact that (1) the endo/lysosome pH in different cells is different and (2) the MB release from the nanoparticles is very sensitive to the endo/lysosomes pH, especially for the NPs_6/100_ and NPs_7.5/80_. From Fig. S23, we can find that nanoparticles have already realized the endocytosis and stayed in the vesicles 2 h after nanoparticle feeding, then nanoparticles gradually accumulated in lysosomes.

**Fig. 5:**
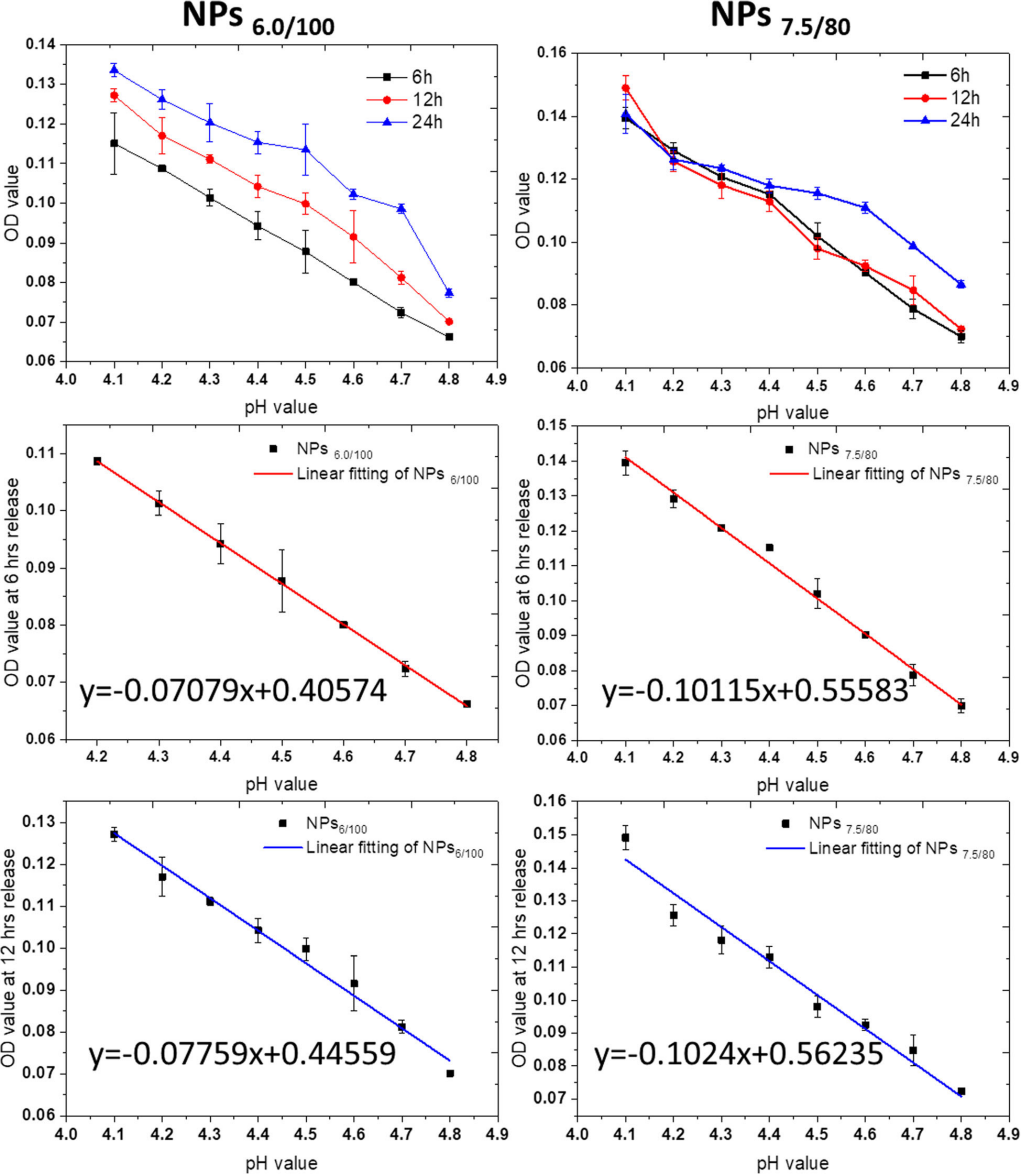
MB release as a function of pH values in NPs6/100 and NPs7.5/80 after specific incubation duration, 6 h, 12 h, and 24 h. The linear correlation equations were also calculated for 6 h and 12 h for MB release from both NPs6/100 and NPs 7.5/80 as a function of pH values. All experiments were carried out triplicated, and the data were shown as mean ± S.D.

We then evaluated the correlation between the pH values and the OD values in pH 4.0–4.8. From the results in Fig. 5 and Fig. S24, for NPs_6/100_ and NPs_7.5/80_ nanoparticle systems, the MB release presented a linear decrease as a function of pH in the pH range from 4.0 to 4.8.

We then converted the OD value to the MB release percentage according to the MB loading efficiency and feeding amount. As shown in Fig. S25, in the first 6 h, the MB release percentage in NPs_6/100_ and NPs_7.5/80_ nanoparticle systems also presented as a function of pH values. We then calculated the residual sum of squares and Pearson’s related coefficient at 6 h and 12 h release duration, respectively, as the residual sum of squares present a negative correlation with closeness of linear fitting, while the closer the absolute value of Pearson’s related coefficient to 1, the more linear it is. As shown in Table S2 and S3, the highest degree of linearity is the fitting of NPs_6.0/100_ nanoparticle systems, followed by the one of NPs_7.5/80_ at 6 h release.

Till then, we were so excited by the results that the method for precisely monitoring the pH values has been established, especially with the accuracy less than or equal to 0.1 pH value interval. That means, we have great possibilities to figure out the correlation between endo/lysosome pH values and the autophagy status, which is of great significance for better studying the autophagy mechanism and predicting the autophagy process. As we can see in Fig. S26, the MB release in HepG-2 cells have already reached the plateau after incubation for 4 h. Thus, we chose 6 h as the observation time point.

We then carefully investigated the MB release of NPs_6.0/100_ in 6 cell lines in the nanoparticle cell interaction duration of 6 h, including liver cancer HepG-2 cell line, colon cancer HCT8, HCT 15, and HCT 116 cell lines, lung cancer A549 cell line, and myomelanocytic cancer B16 cell line.

As shown in Table 2, we clearly differentiate the endo/lysosomes in 6 cancer cell lines, with the accuracy at 0.01 pH values, which is impossible to be done with the commercial intraocular pH indicator kits.

**Table 2:** Endo/lysosomes pH values calculated by equation established in 6 different cell lines

Cell lines	OD values of MB release	pH values calculated by equation ***y*** = − 0.07079***x*** + 0.40574	Errors
**HCT8**	0.066	**4.806**	0.077
**HCT15**	0.111	**4.166**	0.083
**HCT116**	0.081	**4.593**	0.065
**HepG-2**	0.072	**4.708**	0.088
**A549**	0.063	**4.847**	0.078
**B16**	0.062	**4.855**	0.092

Moreover, we re-evaluated pH values in endo/lysosomes of the HepG2 cells before and after cultured with BPSi nanoparticles. We reached the conclusion that BPSi uptake significantly increases the endo/lysosome pH values, from 4.70 ± 0.09 to 5.59 ± 0.05, perfectly illustrating the reason for BPsi uptaken induced the autophagy initially then terminated the autophagy flux. The intracellular uptake of BPSi makes the quantities of the endo/lysosomes increased, which was consistent with the results of gene sequencing, that autophagy-related genes (TFEB-CLEAR) were activated. Meanwhile, the autophagy termination by the increased pH values in endo/lysosomes also coincide with the results of p62 proteins upregulation in Western blot study.

## Discussion

Nanoparticles can generally cause autophagy in cells [23], and studies have shown that the autophagic response to nanoparticles presenting a neutral or anionic surface involves enhanced clearance of autophagic cargo. Cell exposure to nanoparticles presenting a cationic surface, on the other hand, results in transcriptional upregulation of the TFEB pathway, but also causes lysosomal dysfunction, ultimately resulting in blockage of autophagic flux [7]. And our results are in consistent with these previous conclusions. In our study, we found that the expression of autophagy-related genes and proteins in HepG2 cells has been increased after feeding of BPSi nanoparticles through transcriptome sequencing, RT-qPCR, and Western experiments. However, the expression level of autophagy-related P62 protein does not decrease as the autophagy is activated. We suspect that the PEG-amine on the surface of BPSi nanoparticles raises the pH value of the lysosome, resulting in inhibition of P62 degradation. Existing lysosomal pH indicators cannot verify our guess. To accurately measure the lysosomal pH of living cells, we established a new method for endo/lysosomes pH qualitative determination based on self-decomposable nanoparticle systems. Ten nanoparticle systems with specific MB/TEOS parameters were employed for obtaining optimized pH sensitively responsive measurement method. The radial MB concentration gradient from inner out served as a major driving force for MB release. The drug release proceeded with simultaneously carrier decomposition, which was driven by a diffusion-controlled mechanism. Moreover, as the pH value decreases, the hydrogen ion concentration increases, and the enhanced electrostatic interaction promotes inner MB to release faster than in neutral solution [24]. The optimized central hollow nanoparticle system could release the central concentrated MB as a linear function of precise pH values in the range of pH 4.0–4.8, which is exactly the pH of lysosomes. Finally, by this qualitative pH indicator based on self-decomposable nanoparticles, we have succeeded in the detection of the average pH values of lysosomes in 6 cell lines. Moreover, by this system, we can qualitatively differentiate the pH changes of lysosomes before and after BPSi nanoparticle endocytosis by HepG-2 cells, clarifying the mechanism of the autophagy occurrence and then termination after BPSi endocytosis. The self-decomposable nanoparticle systems pave a brand new way for studying the luminal pH values, providing new tools to know better of the cell signaling and metabolism, and then providing new ways and methods for the treatment of cancer [25, 26].

## Conclusion

In this study, we found that BPSi can promote cell autophagy through transcriptome sequencing, but the amino groups on the surface of the nanoparticles can increase the pH of the lysosome and inhibit the degradation of autophagic flow. Thus, the lysosome pH significantly influences the autophagy stages. And precisely acquiring the information of lysosome pH will promote the perceiving of autophagy. However, the existing fluorescent lysosomal pH indicators could only determine a wide range of lysosomal pH; thus, we established a precise lysosomal pH indicator based on the self-dissociation system. By adjusting the synthesis parameters of MB@SiO_2_, the release of MB loaded on the nanoparticles was linearly and negatively correlated with pH. And the nanoparticles mainly stay in the lysosome after entering the cell. By measuring the amount of MB released in the cells, the pH value of the lysosome can be calculated exactly according to the linear function. The established precise pH indicator provided a brand new tool and methodology to precisely study the lysosome pH values and further acquire more information on autophagy.

## Supplementary information

**Additional file 1.** Fig. S1–26 and Table S1–4.

## Data Availability

All data generated or analyzed during this study are included in this published article and its supplementary information files.

## Abbreviations

BPSi: Black mesoporous silicon
FBS: Fetal bovine serum
GO: Gene ontology
HCl: Hydrochloric acid
KEGG: Kyoto Encyclopedia of Genes and Genomes
MB: Methylene blue
NaBr: Sodium bromide
NaSi: Sodium silicide
NH4Br: Ammonium bromide
NPs: Nanoparticles
OD: Optical density
PBS: Phosphate-buffered saline
PDMPO: 2-(4-Pyridyl)-5-((4-(2-dimethylaminoethy- laminocarbamoyl) methoxy) phenyl) oxazole
RT-qPCR: Reverse transcription quantitative polymerase chain reaction
TEOS: Tetraethyl orthosilicate
