# Supplementary material for: The Establishment and Application Studies on Precise Lysosome pH Indicator Based on Self-Decomposable Nanoparticles
Source: Nanoscale Res Lett. 2020 Jul 8;15:143. doi: 10.1186/s11671-020-03367-0 (PMC7343700; doi:10.1186/s11671-020-03367-0)
Supplement: Supplementary file 1 — Additional file 1. Fig. S1–26 and Table S1–4. [file 11671_2020_3367_MOESM1_ESM.docx]

**Supporting information**

**The establishment and application studies on precise lysosome pH indicator based on self-decomposable nanoparticles**

Cui Pang^2#^, Chaojun Song^3#^, Yize Li^2#^, Qiaofeng Wang^1^, Xiaosheng Zhu^2^, Jianwei Wu^2^, Yi Tian^2^, Hao Fan^1^, Jinwei Hu^1^, Chen Li^1^, Baolong Wang^1^, Xiaoye Li^1^, Wenchao Liu ^2*^, Li Fan^1*^

^1^ Department of pharmaceutical analysis, School of Pharmacy, The Fourth military medical university, Xi’an, Shaanxi, China, 710032

^2^ Department of Oncology, Xijing Hospital, The Fourth Military Medical University, Xi’an, 710032, China

^3^ School of Life Science, Northwestern Polytechnic University, 127th Youyi west road, Xi’an, Shaanxi, China, 710072

^*^ Correspondence and requests for materials should be addressed to Fan Li, email: [xxfanny@fmmu.edu.cn](mailto:xxfanny@fmmu.edu.cn) or Wenchao Liu, email: liuch@fmmu.edu.cn

^#^These authors contributed equally to this work.

**
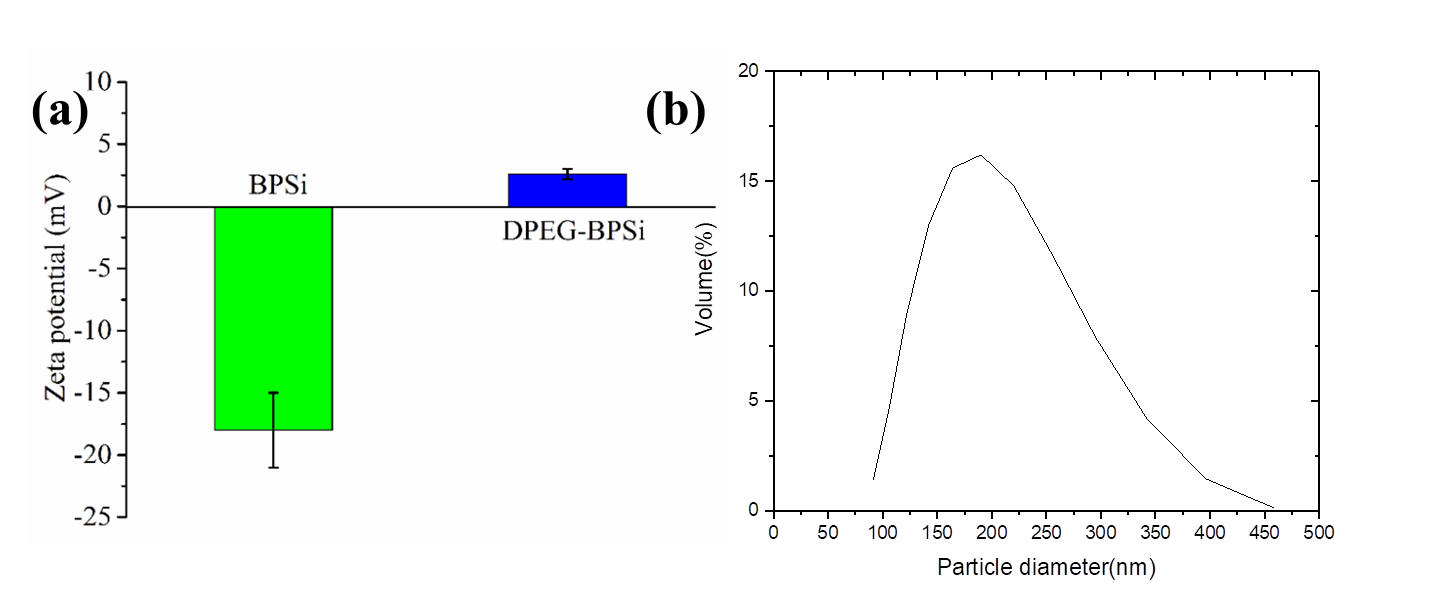
**

**Figure S1.** (a) Zeta potential and (b) particle diameter distribution of BPSi nanoparticles. The surface charges of the nanoparticles were measured in 1:1 diluted PBS at pH 7.4. The nanoparticles were dispersed in deionized H_2_O prior to the measurement.


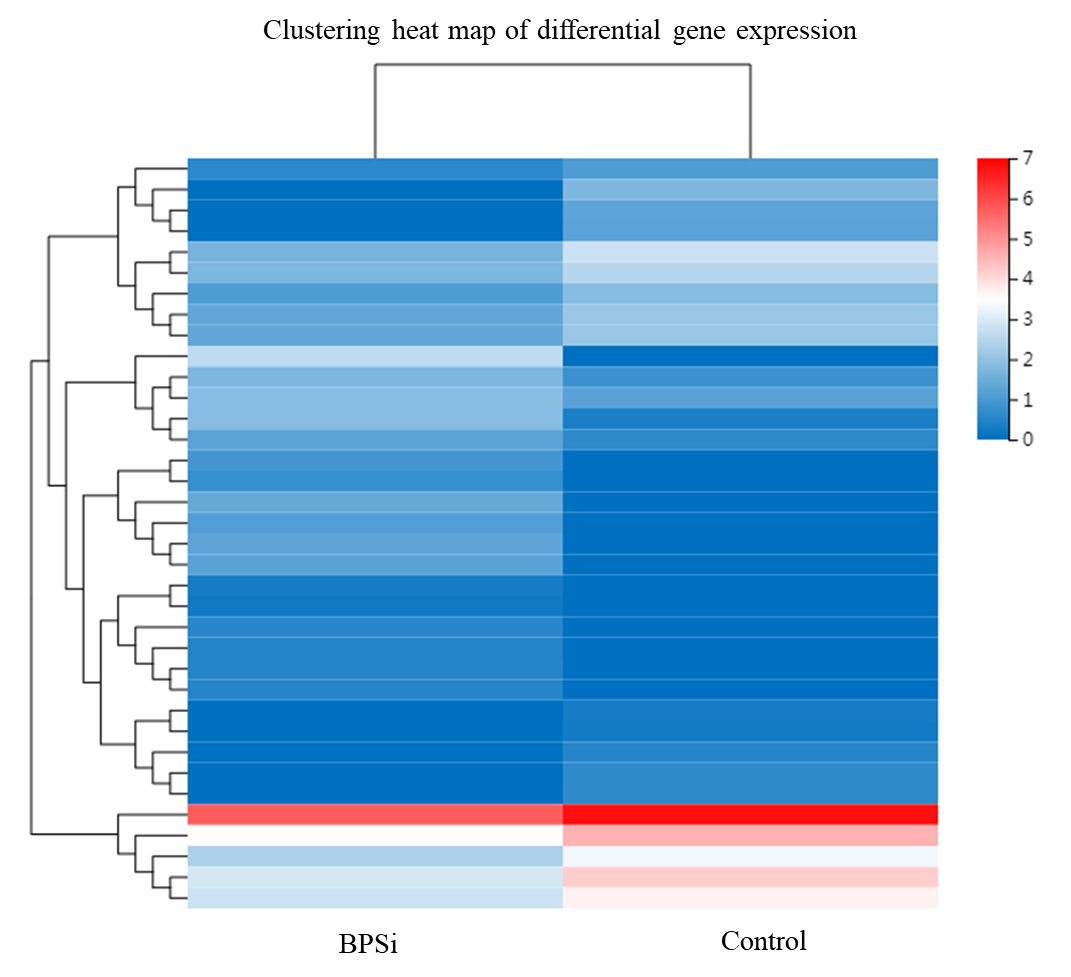


**Figure S2** Cluster heat map of differential gene expression, the scale bar from blue to red represents the quantity of gene differences increase (From 0 to 7 fold).


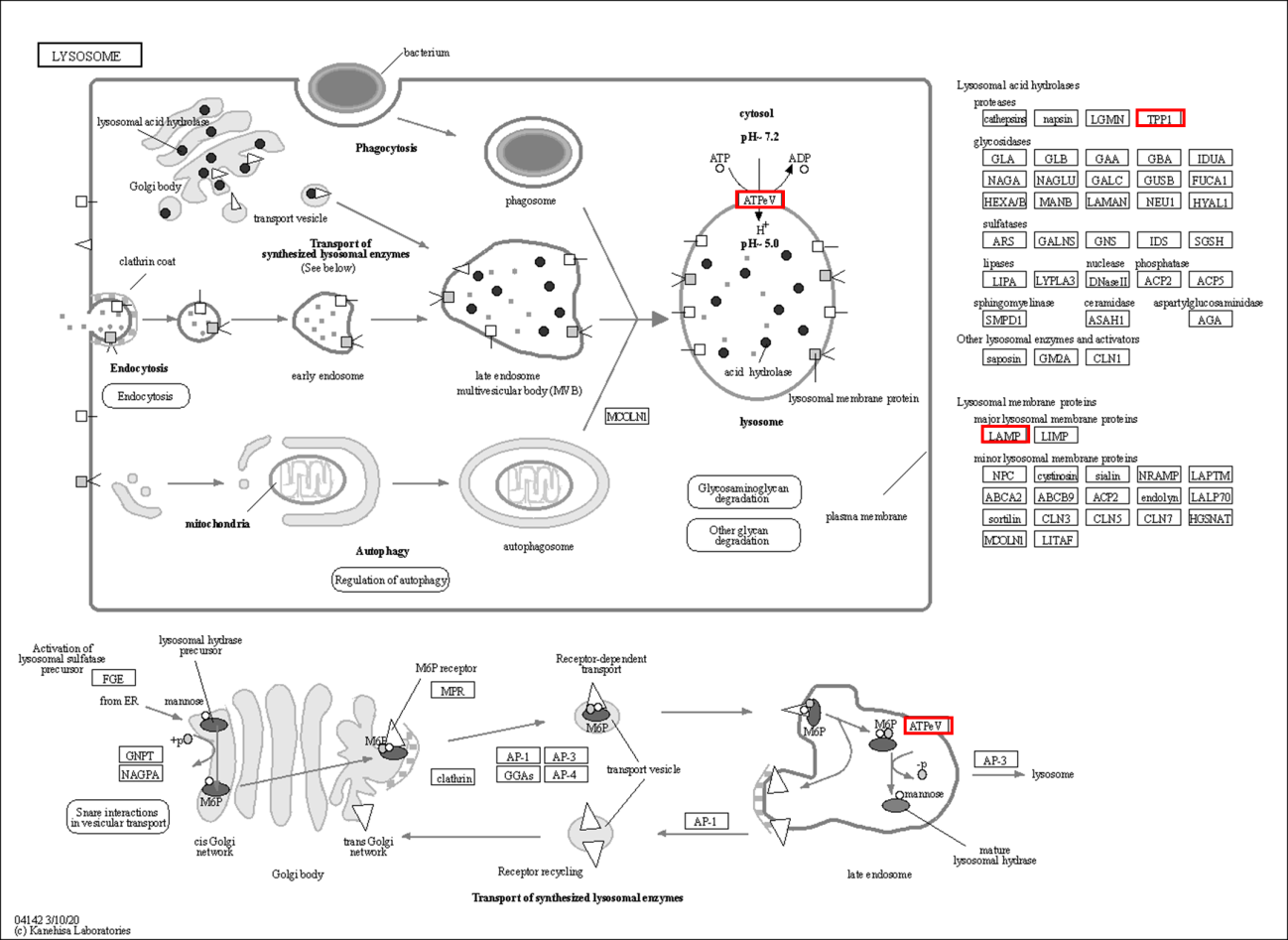


**Figure S3**. Lysosome pathway (map NO. 04142) in KEGG network station. ( https://www.genome.jp/kegg-bin/show_pathway?map04142)


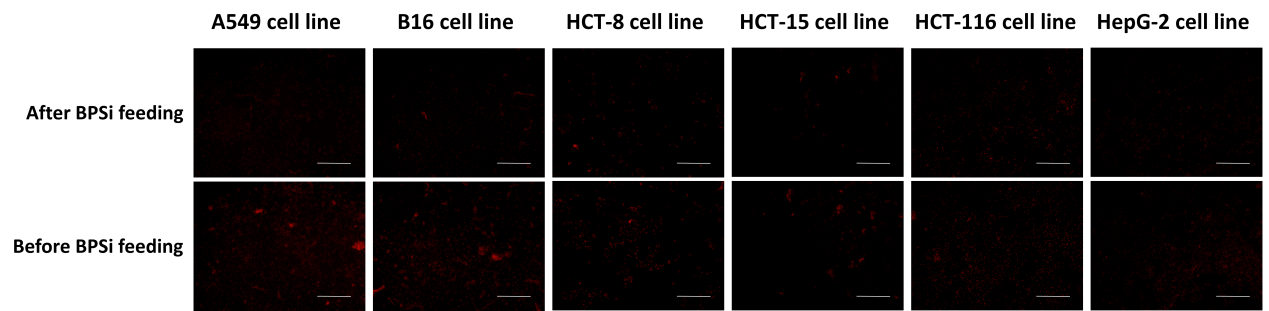


**Figure S4** Intracellular pH measurements in 6 different cell line before and after BPSi nanoparticles feeding. Scale bars in all figures are 100 µm. (200 x magnification)


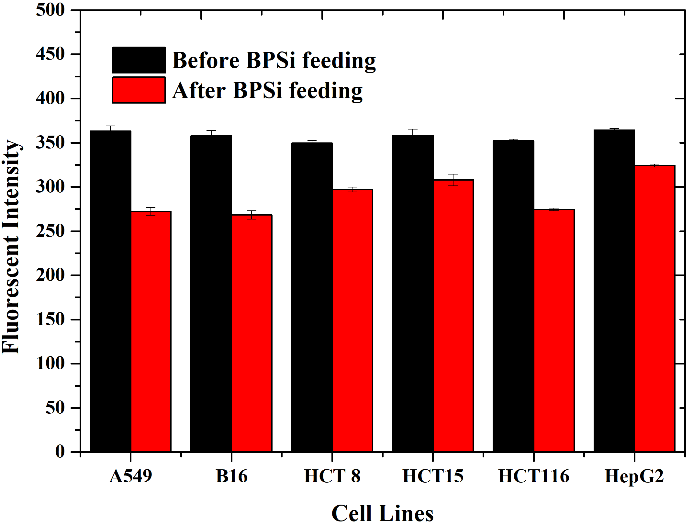


**Figure S5** Drug Quantitative analysis of fluorescent intensity in 6 different cell line before and after BPSi feeding. All experiments were triple repeated, and the data were shown as mean ± S.D..


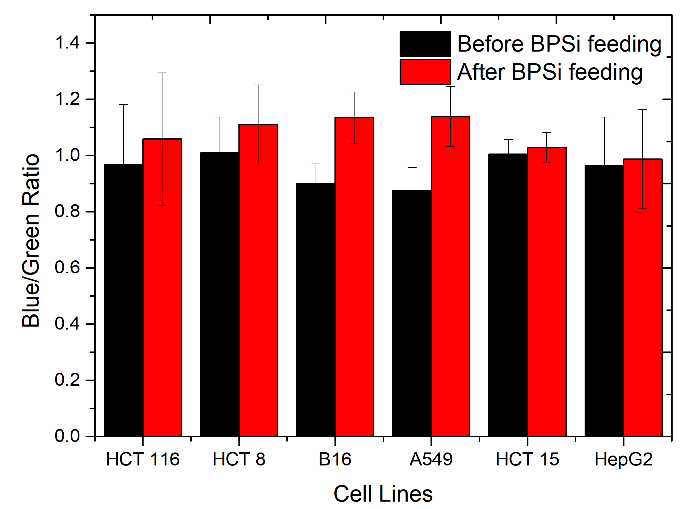


**Figure S6** Drug quantitative analysis of blue and green fluorescent intensity ratio in 6 different cell line before and after BPSi feeding. All experiments were triple repeated, and the data were shown as mean ± S.D..


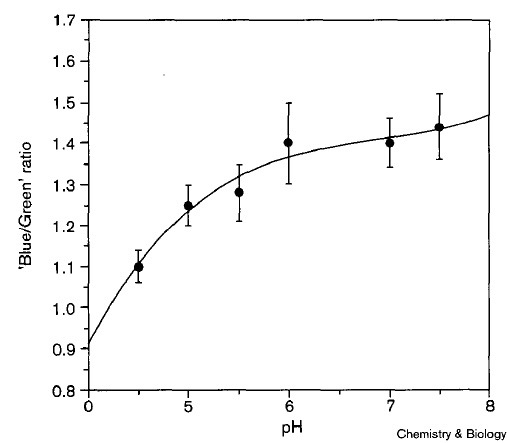


**Figure S7** The correlation between the Blue/Green Ratio and the pH values at the pH range from 0 to 8. (Diwu Z, Chen C-S, Zhang C, Klaubert DH, Haugland RP (1999) A novel acidotropic pH indicator and its potential application in labeling acidic organelles of live cells. Chem Biol 6:411–418.)


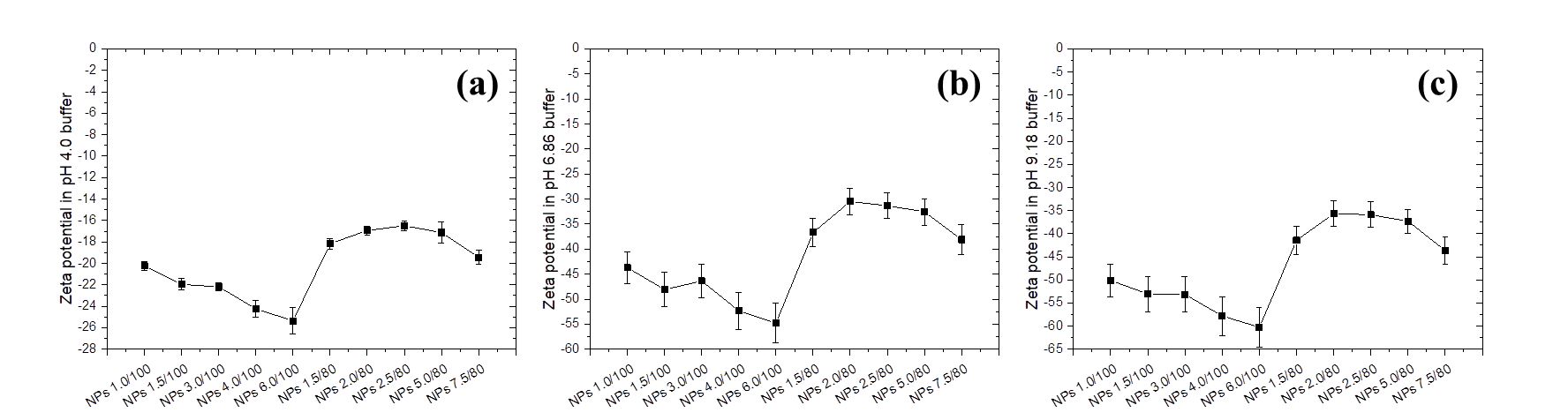


**Figure S8.** Zeta potential of 10 kinds of nanoparticle in standard pH 4.0, pH 6.86 and pH 9.18 buffer.

**Table S1 PDI of 10 kinds of nanoparticle in standard pH 4.0, pH 6.86 and pH 9.18 buffer.**

|  | PDI in pH 4.14 buffer | PDI in pH 6.86 buffer | PDI in pH 9.18 buffer |
| --- | --- | --- | --- |
| NPs 1.0/100 | 0.11±0.0015 | 0.29±0.016 | 0.36±0.027 |
| NPs 1.5/100 | 0.093±0.0035 | 0.31±0.024 | 0.31±0.015 |
| NPs 3.0/100 | 0.13±0.0019 | 0.24±0.049 | 0.34±0.025 |
| NPs 4.0/100 | 0.101±0.0075 | 0.29±0.013 | 0.38±0.0396 |
| NPs 6.0/100 | 0.076±0.0016 | 0.214±0.0098 | 0.349±0.014 |
| NPs 1.5/80 | 0.105±0.0024 | 0.284±0.0014 | 0.247±0.026 |
| NPs 2.0/80 | 0.049±0.0059 | 0.219±0.015 | 0.354±0.078 |
| NPs 2.5/80 | 0.103±0.0084 | 0.197±0.046 | 0.376±0.045 |
| NPs 5.0/80 | 0.112±0.0069 | 0.196±0.027 | 0.319±0.025 |
| NPs 7.5/80 | 0.167±0.016 | 0.179±0.096 | 0.29±0.049 |


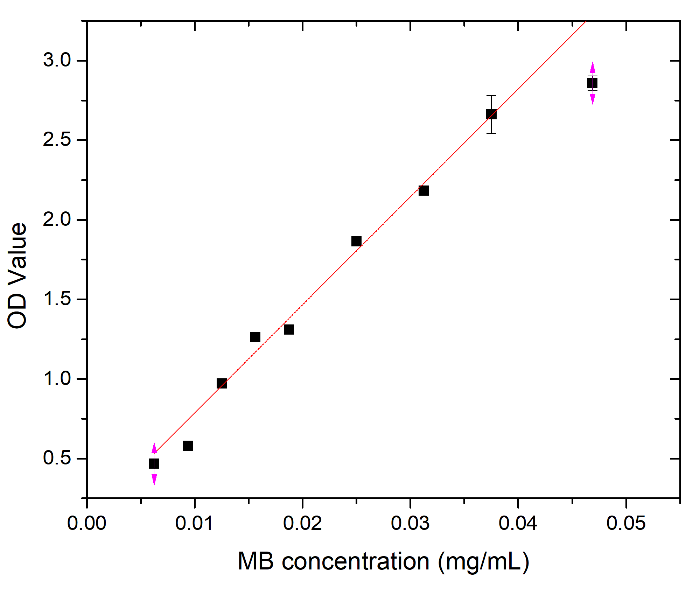


**Figure** **S9** Standard curve of MB solution at series MB concentration.


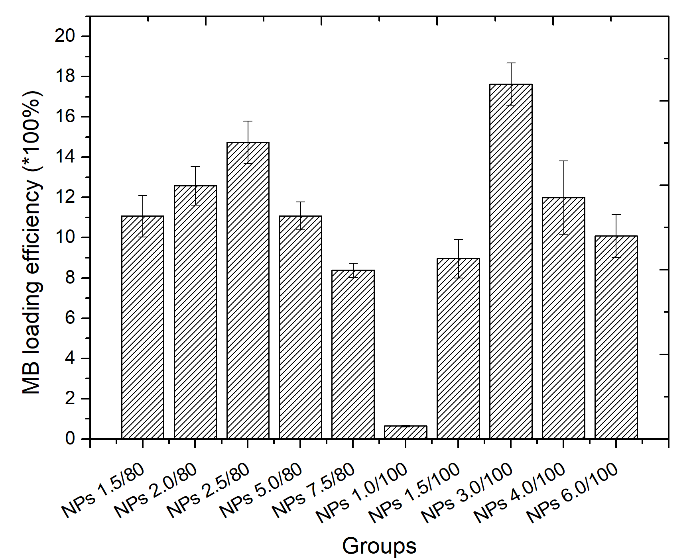


**Figure S10** MB loading efficiency of 10 self-decomposable nanoparticles with specific parameters. All experiments were triple repeated, and the data were shown as mean ± S.D..


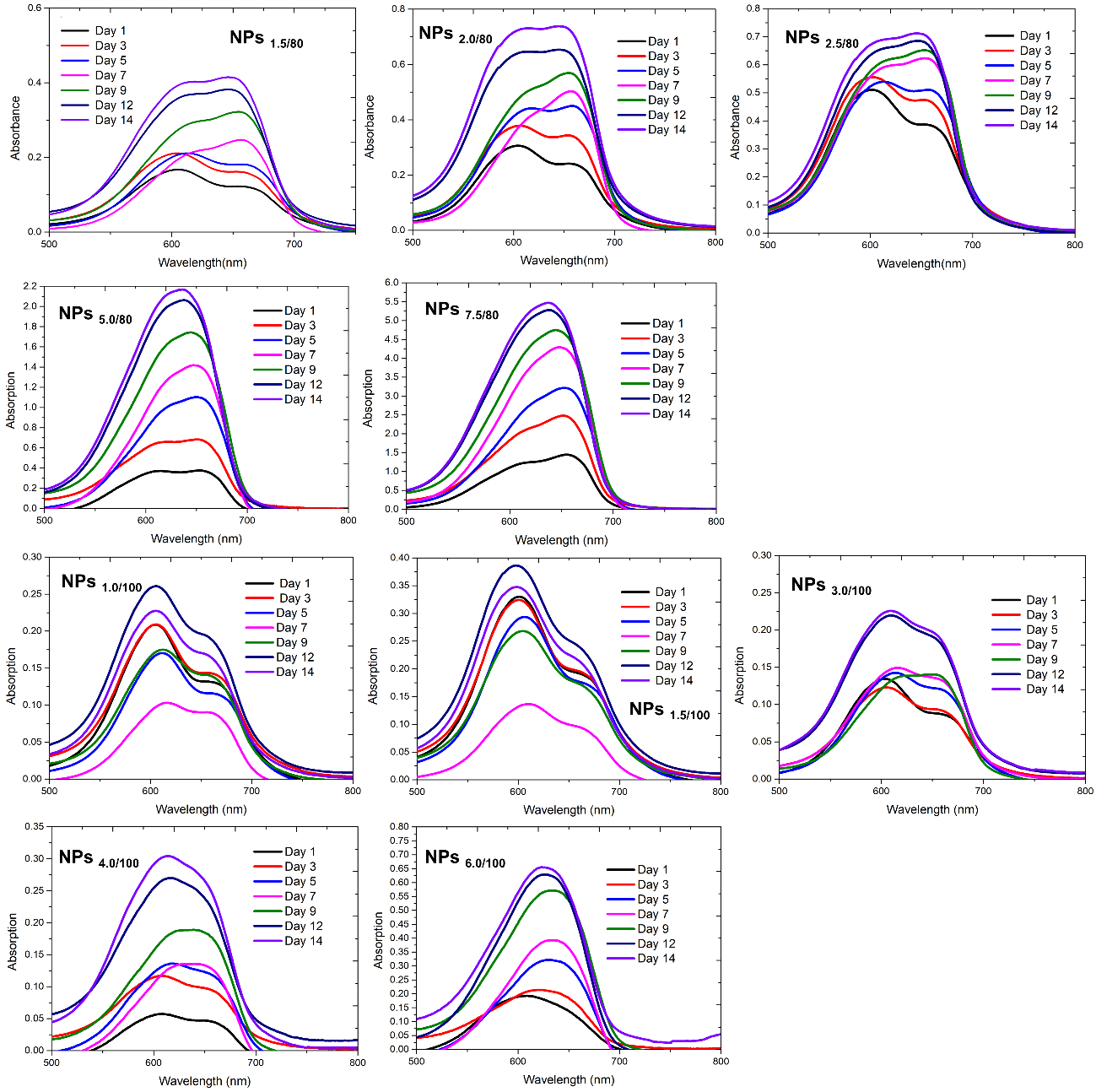


**Figure S11** Absorption of MB release from 10 self-decomposable nanoparticles with specific parameters were recorded with the spectrum range from 500-800 nm during 14 days immersed in distilled water.


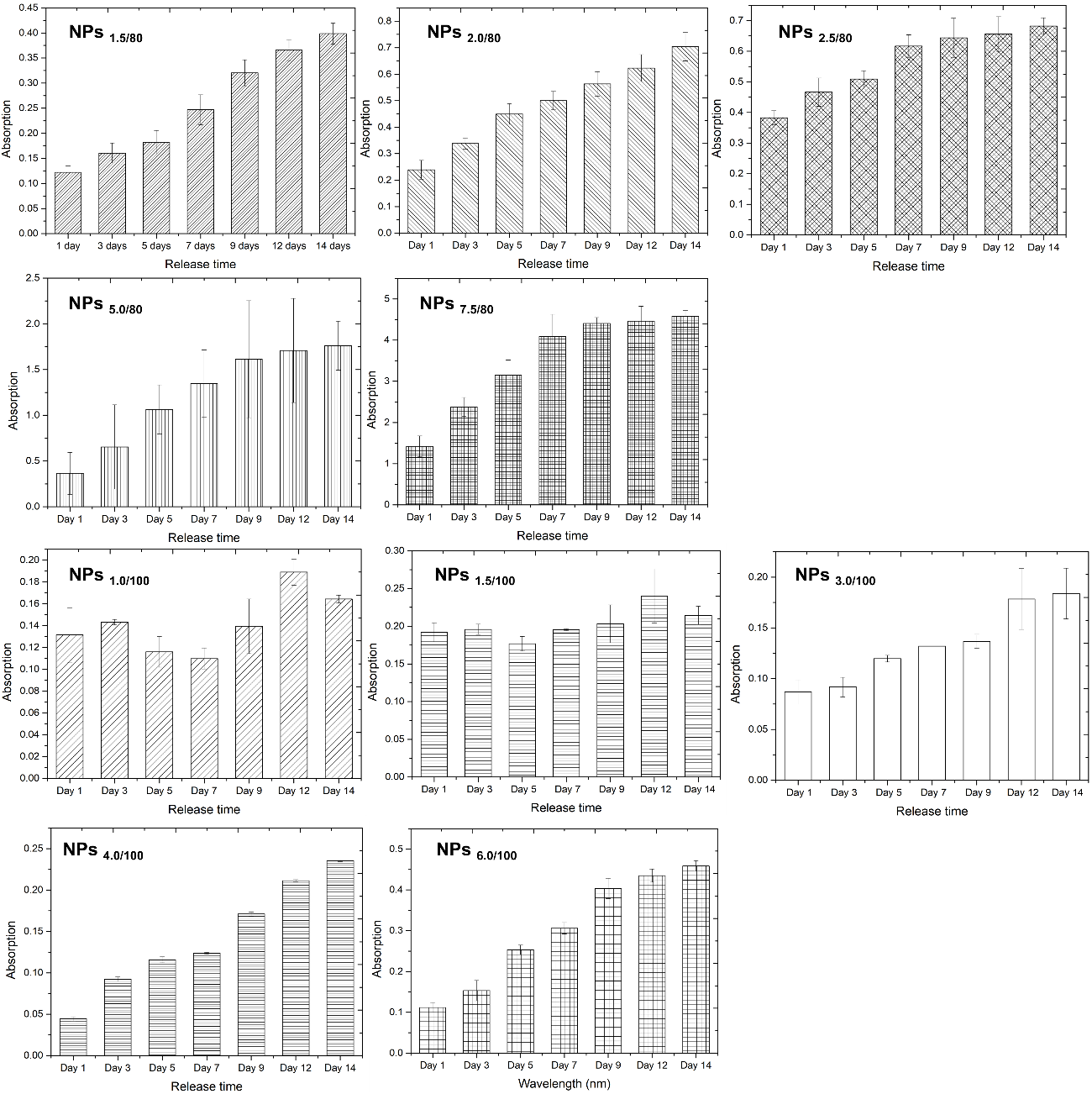


**Figure S12** Peak value at 660 nm in specific time duration was selected in 10 series of nanoparticles to give more direct evidence on MB release profiles. All experiments were triple repeated, and the data were shown as mean ± S.D..


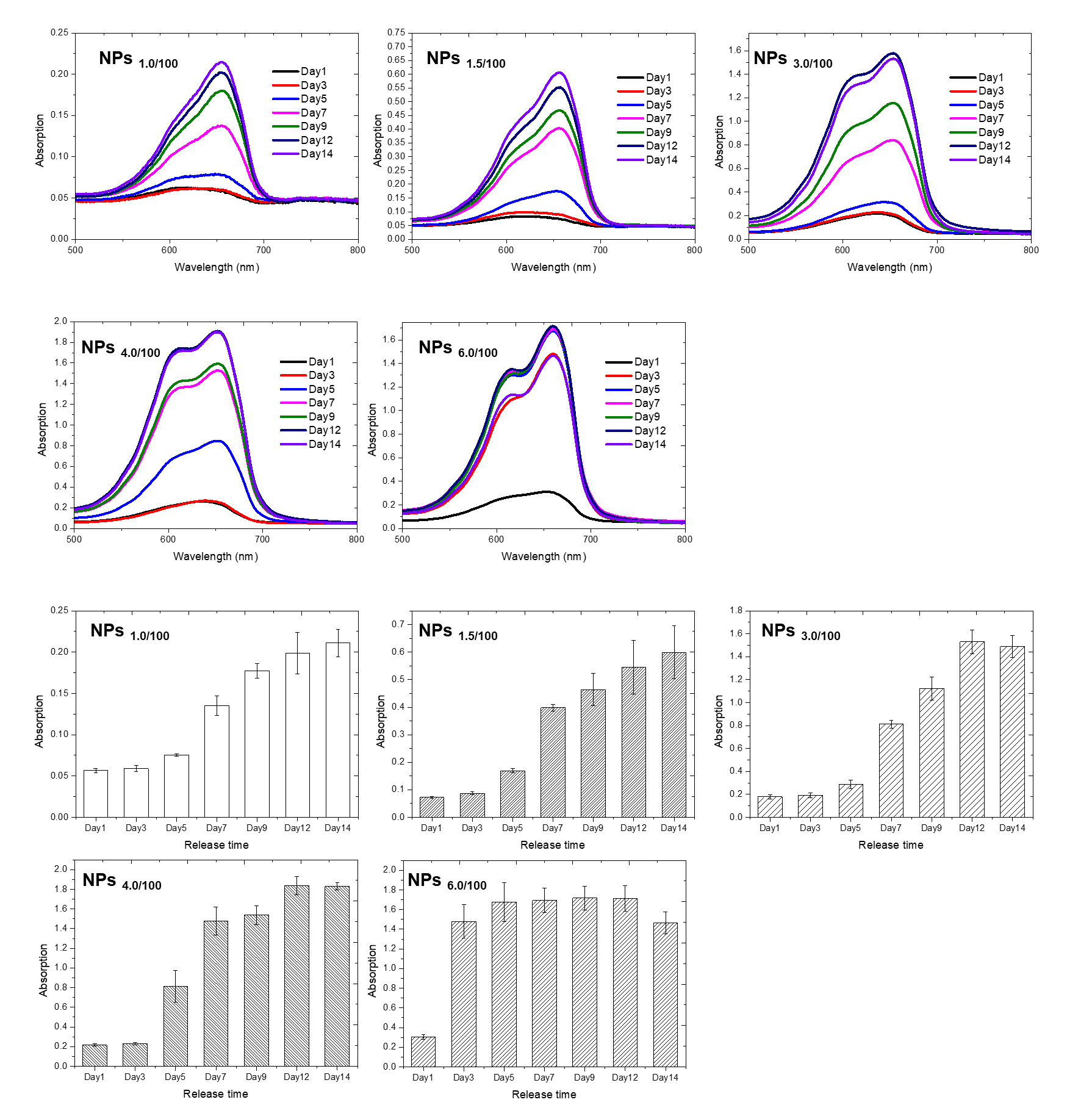


**Figure S13** MB release profile of 5 self-decomposable nanoparticles in pH 4 buffer solution after specific duration. UV-Vis spectrums and MB release profiles of 5 series self-decomposable nanoparticles with TEOS adding amount of 100 μL. All experiments were triple repeated, and the data were shown as mean ± S.D..


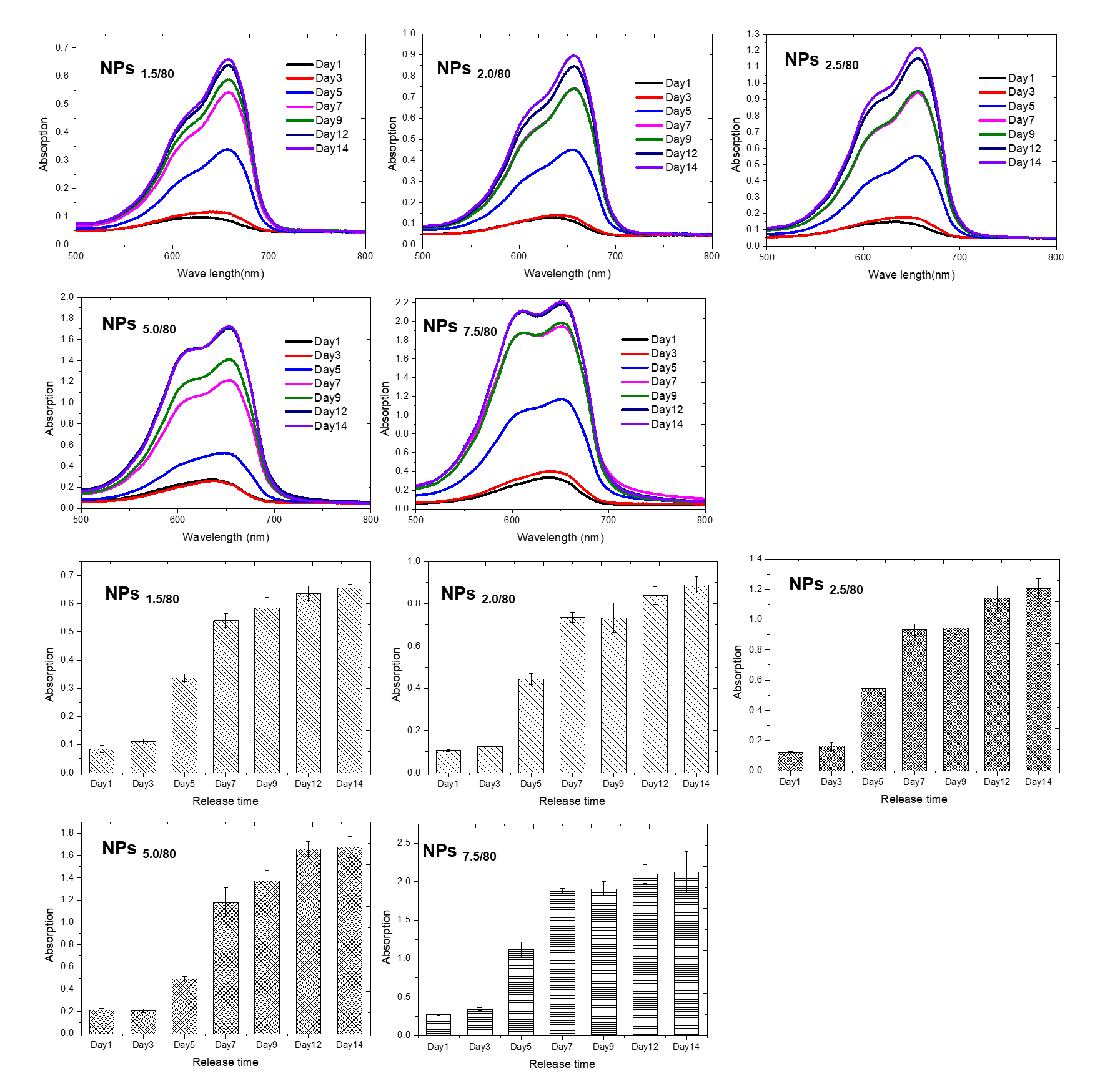


**Figure S14** MB release profile of 5 self-decomposable nanoparticles in pH 4 buffer solution as a function of release duration. UV-Vis spectrums and MB release profiles of 5 series self-decomposable nanoparticles with TEOS adding amount of 80 μL. All experiments were triple repeated, and the data were shown as mean ± S.D..


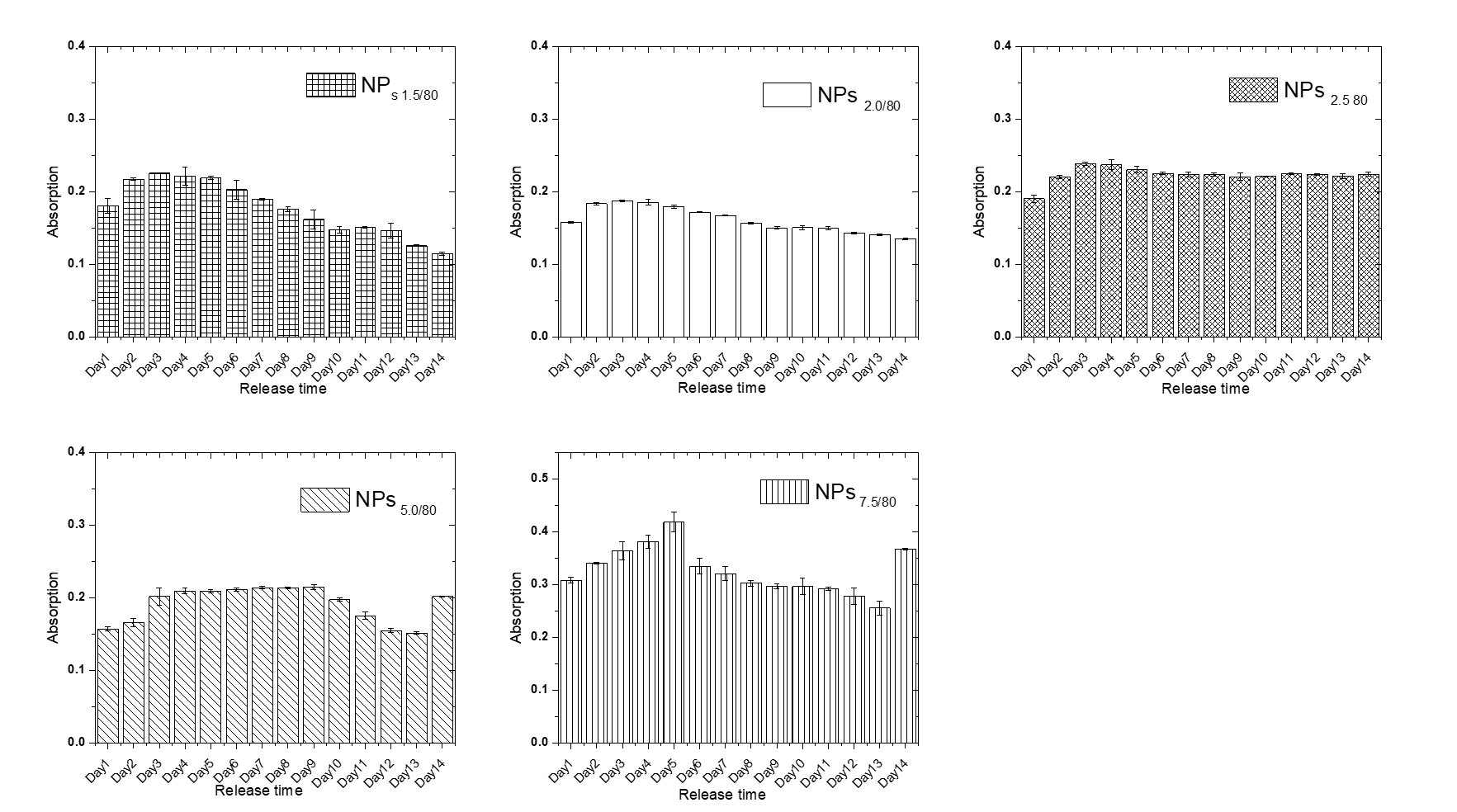


**Figure S15** MB release profile by UV-Vis spectrums of 5 self-decomposable nanoparticles with TEOS adding amount of 80 μL in pH 6.86 buffer solution as a function of release duration. All experiments were triple repeated, and the data were shown as mean ± S.D..


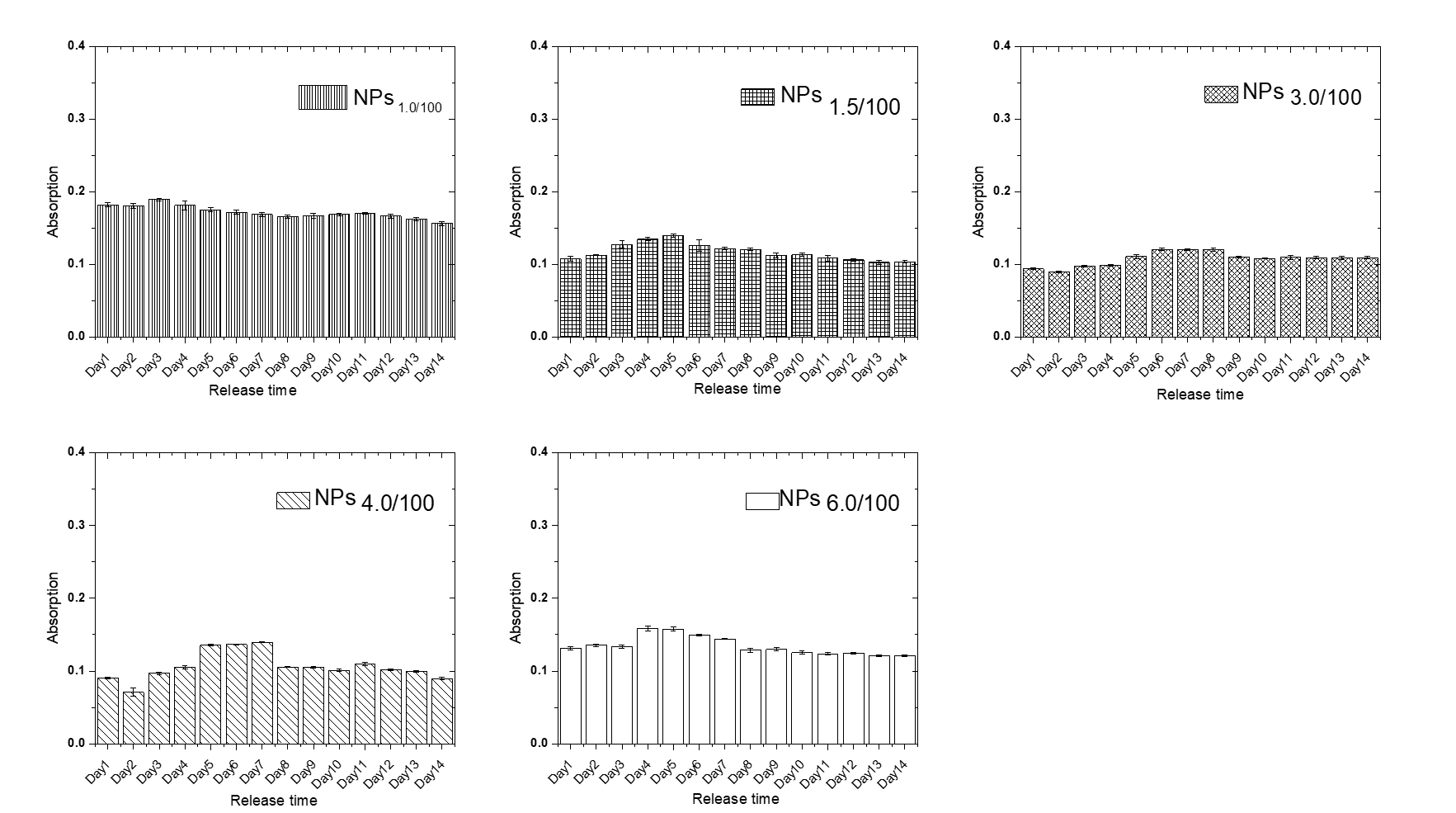


**Figure S16** MB release profile by UV-Vis spectrums of 5 self-decomposable nanoparticles with TEOS adding amount of 100 μL in pH 6.86 buffer solution as a function of release duration. All experiments were triplicated, and the data were shown as mean ± S.D..


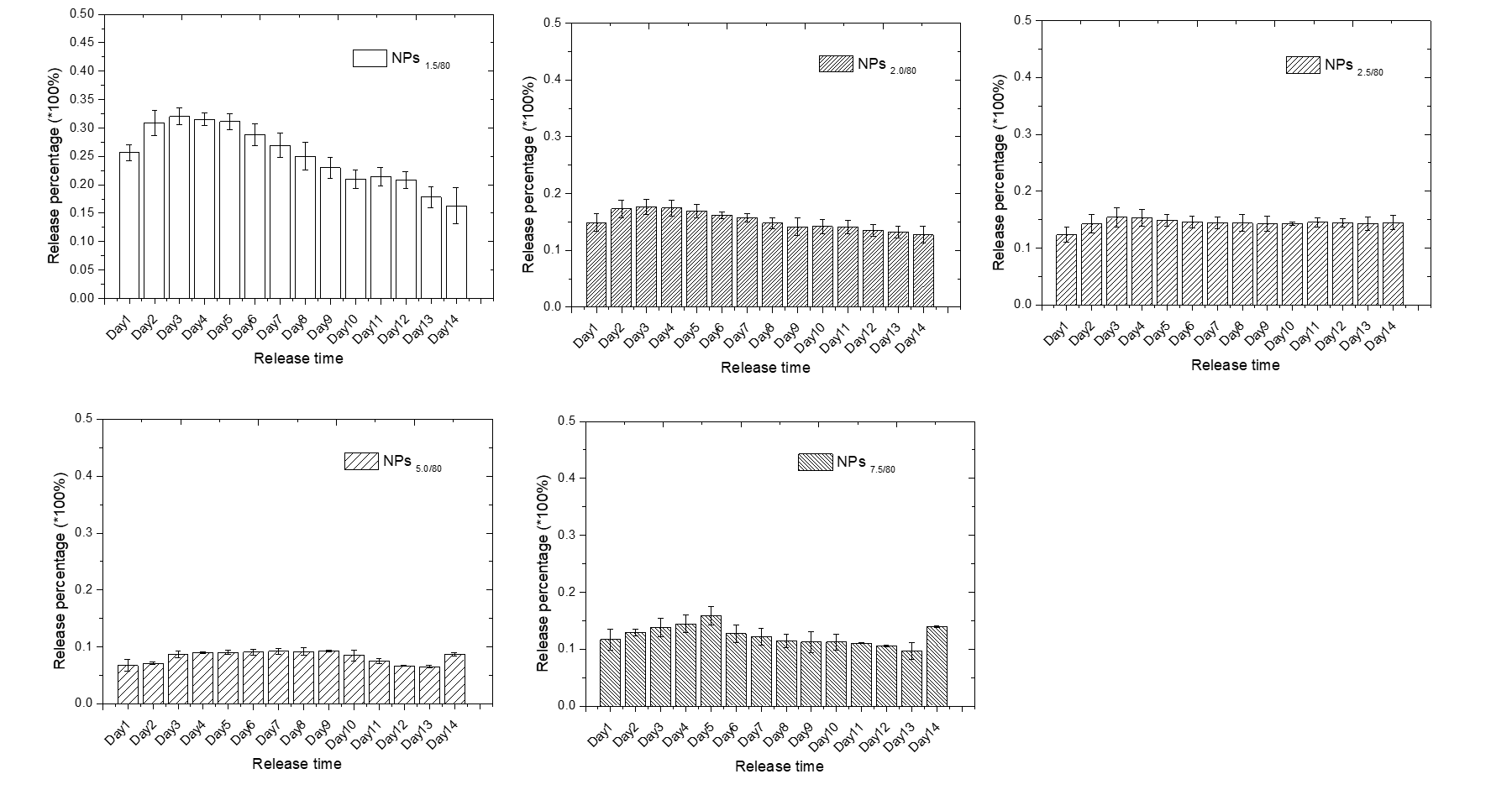


**Figure S17** MB release percentage profiles of 5 self-decomposable nanoparticles with TEOS adding amount of 80 μL in pH 6.86 buffer solution as a function of release time duration. All experiments were triplicated, and the data were shown as mean ± S.D..


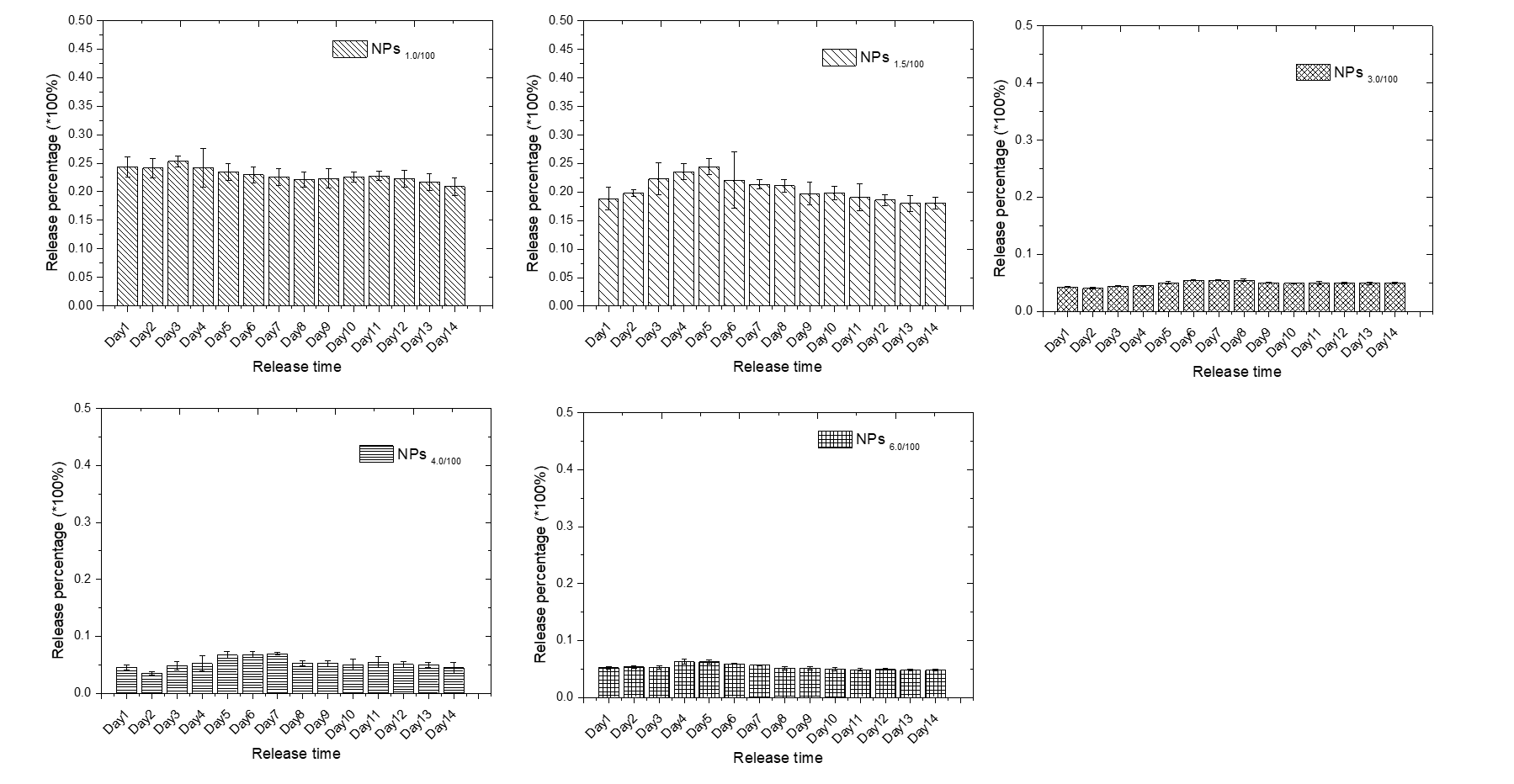


**Figure S18** MB release percentage profiles of 5 self-decomposable nanoparticles with TEOS adding amount of 100 μL in pH 6.86 buffer solution as a function of release time duration. All experiments were triplicated, and the data were shown as mean ± S.D..


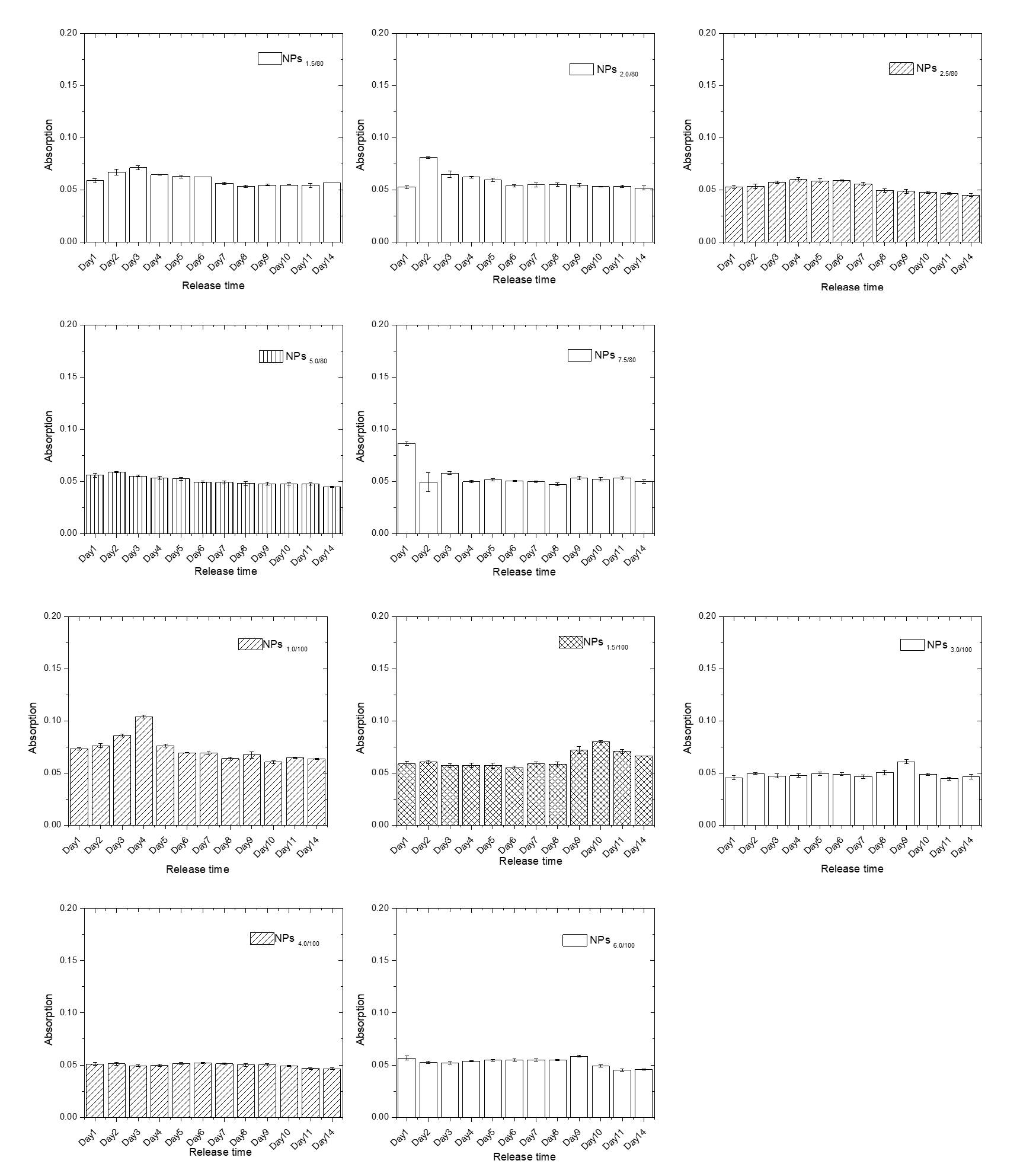


**Figure S19** MB release profile by UV-Vis spectrums of 10 self-decomposable nanoparticles with TEOS adding amount of 80 μL and 100 μL in pH 9.18 buffer solution as a function of release duration. All experiments were triplicated, and the data were shown as mean ± S.D..


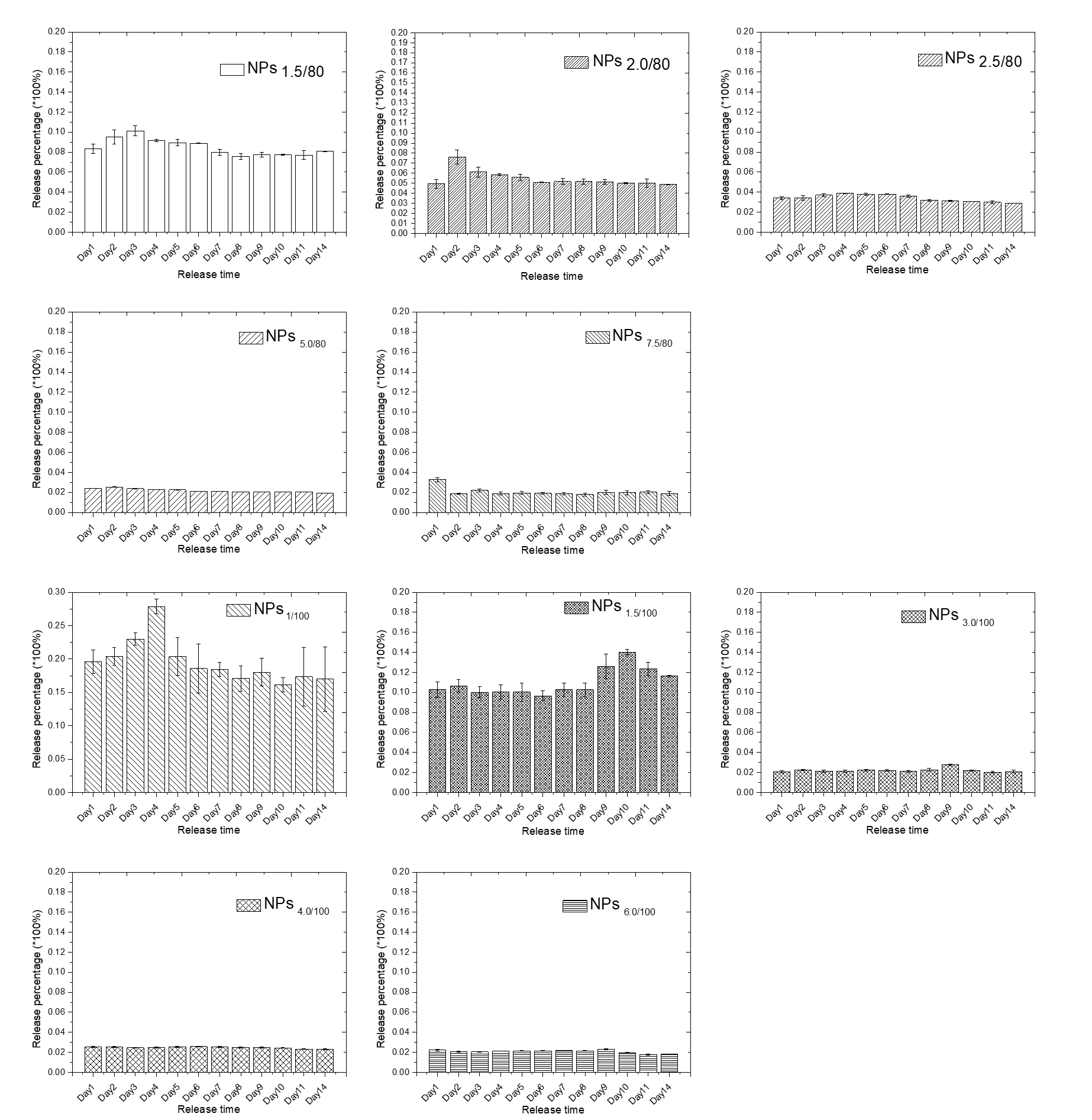


**Figure S20** MB release percentage profiles of 10 self-decomposable nanoparticles with TEOS adding amount of 80 μL and 100 μL in pH 9.18 buffer solution as a function of release time duration. All experiments were triplicated, and the data were shown as mean ± S.D..


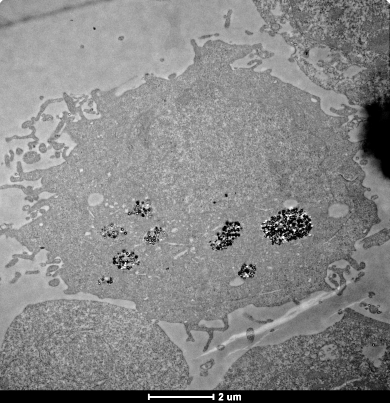
Figure S21. The colocalization of the nanoparticle in the endo/lysosomes by cell TEM study after 24 hrs incubation with the NPs _2.5/80_. The scale bar is 2 µm in the TEM images.


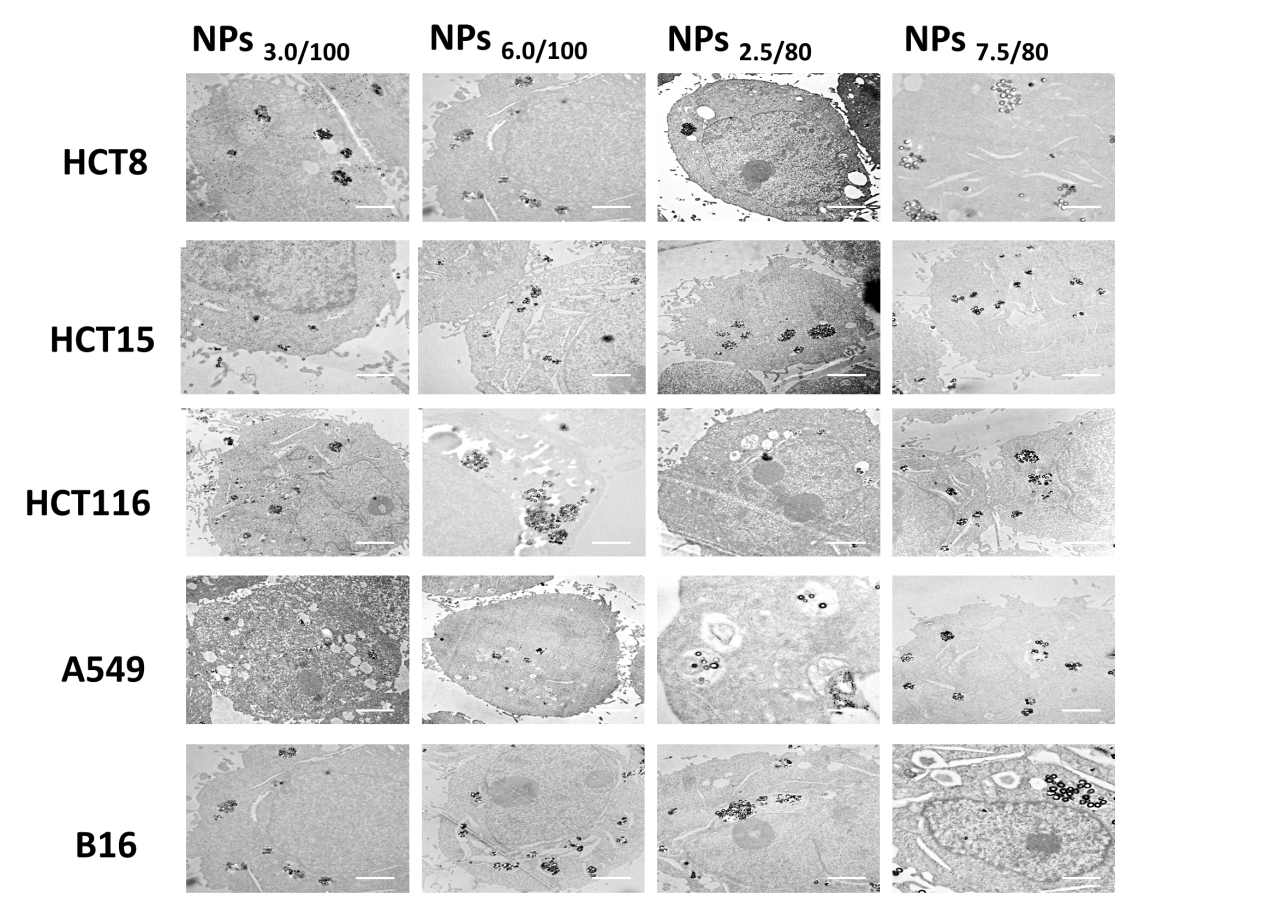


**Figure S22** The colocalization of the nanoparticle in the endo/lysosomes by cell TEM study after 24 hrs incubation with the randomly chosen of nanoparticles of both 80 μL and 100 μL TEOS amount groups in HCT8, HCT15, HCT116, A549 and B16 cell lines. The scale bar is 1 µm in the TEM images.


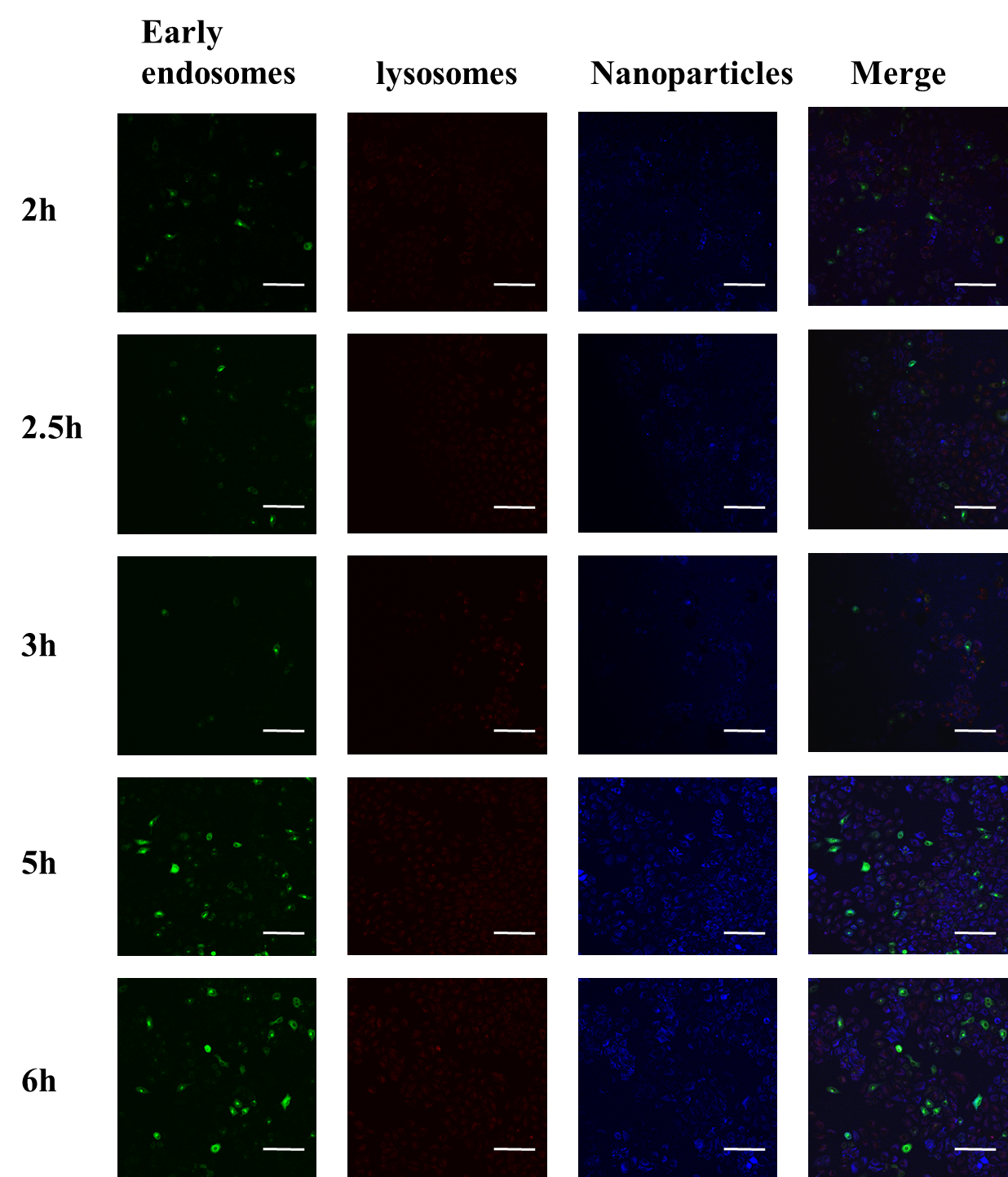


**Figure S23** The colocalization of the nanoparticle in the early endosomes or lysosomes by cell confocal study after several time point incubation with the NPs _6.0/100_ in HepG-2 cell lines. Scale bars in all figures are 100 µm. (200 x magnification)


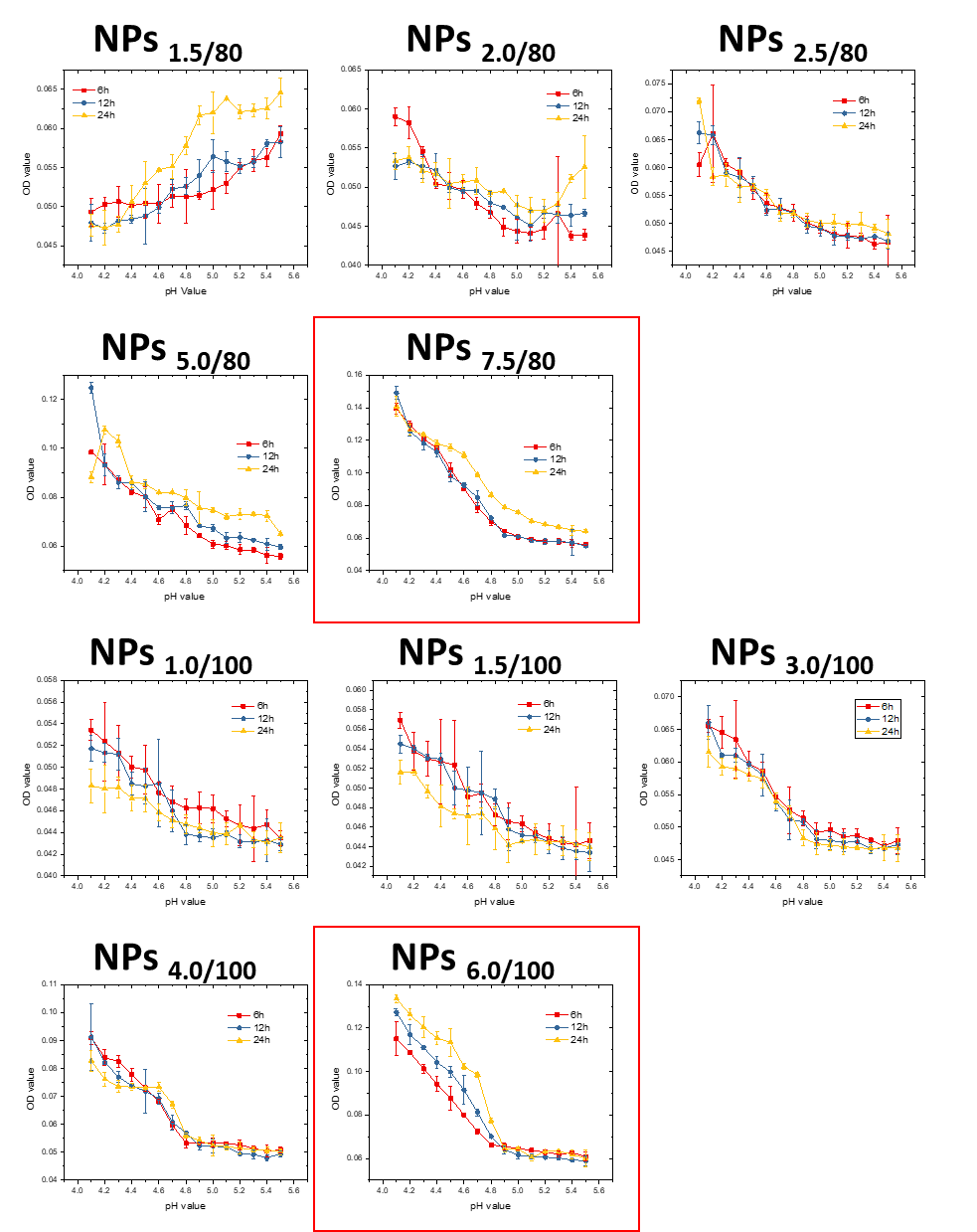


**Figure S24** MB release as a function of pH values in 10 series self-decomposable nanoparticles after specific incubation duration, 6 hrs, 12 hrs and 24 hrs. All experiments were carried out triplicated, and the data were shown as mean ± S.D..


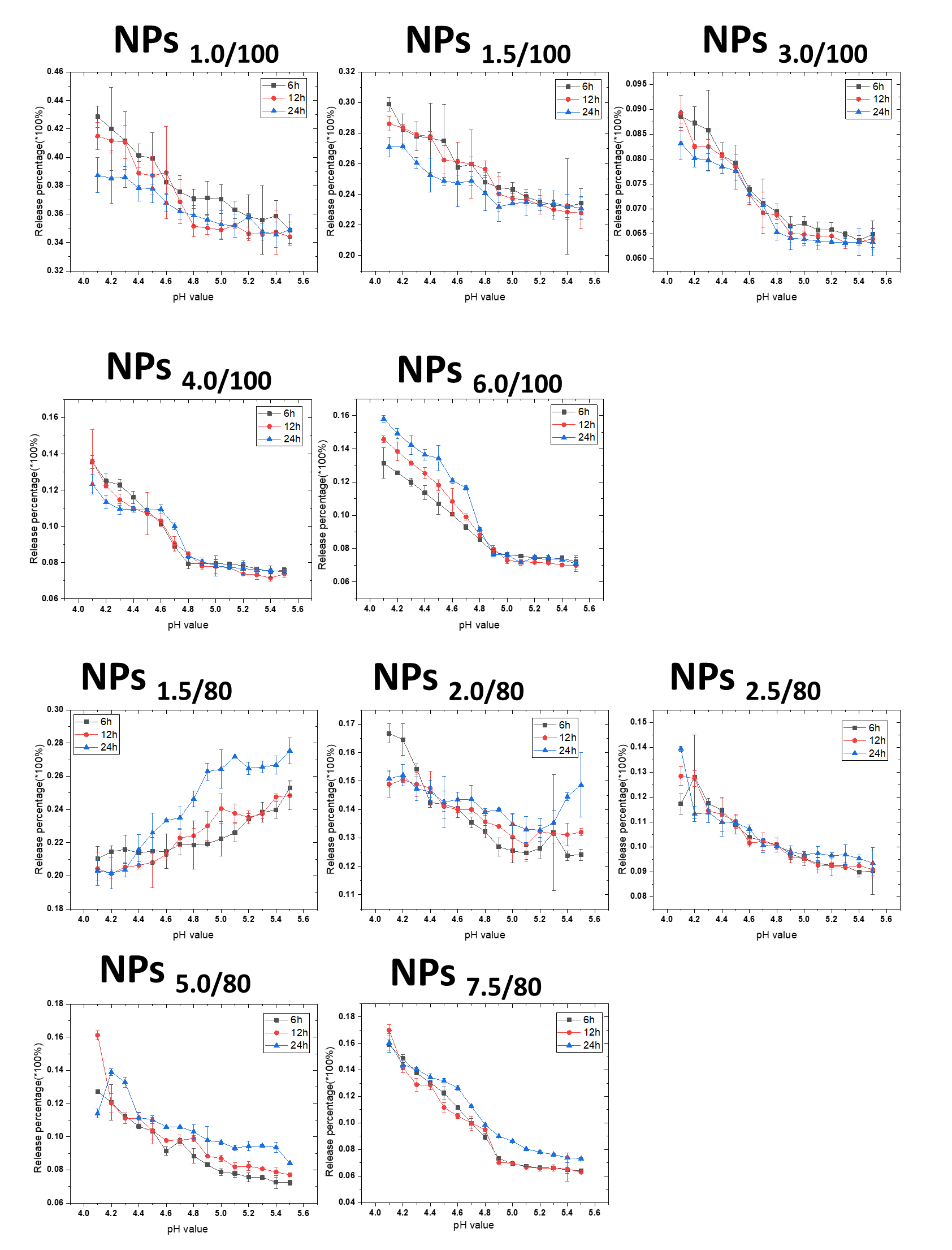


**Figure S25** MB release percentage as a function of pH values in 10 series self-decomposable nanoparticles as a function of pH values, 6 hrs, 12 hrs and 24 hrs. All experiments were carried out triplicated, and the data were shown as mean ± S.D..

**Table S2** Residual Sum of Squares and Pearson's related coefficient at 6 hrs release duration of 10 nanoparticle systems linear fitting

|  | NPs _1.5/80_ | NPs _2.0/80_ | NPs _2.5/80_ | NPs _5.0/80_ | NPs _7.5/80_ | NPs _1.0/100_ | NPs _1.5/100_ | NPs _3.0/100_ | NPs _4.0/100_ | NPs _6.0/100_ |
| --- | --- | --- | --- | --- | --- | --- | --- | --- | --- | --- |
| Residual Sum of Squares | 0.09948 | 0.03363 | 0.07444 | 0.02647 | 0.01776 | 0.00595 | 0.03147 | 0.00938 | 0.00973 | 0.01471 |
| Pearson's r | 0.82369 | -0.95903 | -0.90264 | -0.96792 | -0.98960 | -0.9827 | -0.9602 | -0.9885 | -0.98833 | -0.99223 |





**Figure S26** MB release amount in HepG-2 cells after incubation different times. All experiments were carried out triplicated, and the data were shown as mean ± S.D..

**Table S3** Residual Sum of Squares and Pearson's related coefficient at 12 hrs release duration of 10 nanoparticle systems linear fitting

|  | NPs _1.5/80_ | NPs _2.0/80_ | NPs _2.5/80_ | NPs _5.0/80_ | NPs _7.5/80_ | NPs _1.0/100_ | NPs _1.5/100_ | NPs _3.0/100_ | NPs _4.0/100_ | NPs _6.0/100_ |
| --- | --- | --- | --- | --- | --- | --- | --- | --- | --- | --- |
| Residual Sum of Squares | 0.06116 | 0.04536 | 0.0339 | 0.13642 | 0.05542 | 0.03859 | 0.02997 | 0.01969 | 0.02581 | 0.0148 |
| Pearson's r | 0.91893 | -0.94261 | -0.9587 | -0.82146 | -0.9316 | -0.95015 | -0.96268 | -0.97613 | -0.96874 | -0.98198 |

**Table S4**. Primers used for quantitative RT-PCR

| Gene Name | Forward primer | Reverse primer |
| --- | --- | --- |
| MFN1 | TGGCTAAGAAGGCGATTACTGC | TCTCCGAGATAGCACCTCACC |
| MFN2 | CTCTCGATGCAACTCTATCGTC | TCCTGTACGTGTCTTCAAGGAA |
| OPA1 | TGTGAGGTCTGCCAGTCTTTA | TGTCCTTAATTGGGGTCGTTG |
| DRP1 | CTGCCTCAAATCGTCGTAGTG | GAGGTCTCCGGGTGACAATTC |
| TFEB | CCAGAAGCGAGAGCTCACAGAT | TGTGATTGTCTTTCTTCTGCCG |
| CTSA | CAGGCTTTGGTCTTCTCTCCA | TCACGCATTCCAGGTCTTTG |
| CTSB | AGTGGAGAATGGCACACCCTA | AAGAAGCCATTGTCACCCCA |
| CTSD | AACTGCTGGACATCGCTTGCT | CATTCTTCACGTAGGTGCTGGA |
| CTSF | ACAGAGGAGGAGTTCCGCACTA | GCTTGCTTCATCTTGTTGCCA |
| GNS | CCCATTTTGAGAGGTGCCAGT | TGACGTTACGGCCTTCTCCTT |
| TPP1 | GATCCCAGCTCTCCTCAATACG | GCCATTTTTGCACCGTGTG |
| MCOLN1 | TTGCTCTCTGCCAGCGGTACTA | GCAGTCAGTAACCACCATCGGA |
| LAMP1 | ACGTTACAGCGTCCAGCTCAT | TCTTTGGAGCTCGCATTGG |
| LAMP2 | GCACAGTGAGCACAAATGAGT | CAGTGGTGTGTATGGTGGGT |
| ATP6VOD2 | TCTCACCTATATGACGTGCAGT | GGTGGCACTTCCCCAGAATTT |
| ATP6V0E1 | CATTGTGATGAGCGTGTTCTGG | AACTCCCCGGTTAGGACCCTTA |
| ATP6V1H | GGAAGTGTCAGATGATCCCCA | CCGTTTGCCTCGTGGATAAT |
| CLCN7 | TGATCTCCACGTTCACCCTGA | TCTCCGAGTCAAACCTTCCGA |
| ACTIN | CTGTCCACCTTCCAGCAGATGT | CGCAACTAAGTCATAGTCCGCC |

The specific steps for preparing the cell TEM are as follows:

To prepare the cell samples for TEM study, the cells after NPs treatment were detached from the bottom of flasks by trypsin digestion and fixed in 2.5% glutaraldehyde at 4^o^C overnight. Then the cells were washed by phosphate buffered saline (PBS) for at least two times, and post-fixed in 1% osmium tetroxide (OsO_4_) PBS solution for 1 hour at room temperature, following by centrifuging the mixture at 3000 rpm for 5 min, discarding the OsO4 supernatant and washing cells with PBS for at least two times. The cells were then immersed into 2% agar solution and centrifuged immediately at 7500 rpm for 10 min. Cell cubes were collected and dehydrated in ethanol. Dehydration process was carried out on a rotary shaker as follows:

50% ethanol - 10 min room temperature (RT)

70% ethanol - 10 min RT

90% ethanol - 10 min RT

100% ethanol - 3 changes, 20 min each RT

Propylene oxide - 2 changes, 10 min each RT

Then the cell cubes were infiltrated with epoxy resin(spurr):propylene oxide mixtures:

1:2 mixture - 1.5 h 37 ℃

1:1 mixture - 1.5 h 37 ℃

2:1 mixture - overnight RT (in drying box)

After that, the cell cubes were transfered to fresh epoxy resin(spurr) contained in dried, labeled bee-capsules and polymerized at 60℃ overnight. Microtome (Leica, EM UC6) was then used to cut the cured cell cube into nano-scale thin slices (70-90 nm in thickness). The tiny slices (about 0.5 mm×0.5 mm) were collected on 300-mesh copper grids for TEM observation.
